# Comparative Analysis of Japanese Clinical Note Styles Between Physicians and Large Language Models Using Identical Psychiatric Cases: Quantitative Text Analysis

**DOI:** 10.2196/85671

**Published:** 2026-03-27

**Authors:** Wataru Arihisa, Tomohiro Nishiyama, Shoko Wakamiya, Eiji Aramaki

**Affiliations:** 1 Department of Information Science, Graduate School of Science and Technology, Nara Institute of Science and Technology, 8916-5 Takayama-cho, Ikoma, 630-0192, Japan, 81 0743-72-5111

**Keywords:** large language models, clinical documentation, psychiatry, natural language processing, medical informatics

## Abstract

**Background:**

With the rapid adoption of large language models (LLMs) in clinical documentation, it is unclear whether LLMs can faithfully reproduce specialty-specific writing styles and clinically meaningful documentation patterns observed in expert notes, particularly in psychiatry.

**Objective:**

This study aims to systematically compare the narrative styles of human physicians and LLMs when documenting identical psychiatric cases and to evaluate the extent to which LLMs replicate specialty-specific documentation patterns.

**Methods:**

We constructed 2 standardized outpatient scenarios in Japanese (major depressive disorder and schizophrenia) and collected 134 initial notes in Japanese authored by psychiatrists and internists, alongside notes generated by 4 LLMs simulating each specialty. We conducted lexical, syntactic, semantic, and topic-level analyses using Bilingual Evaluation Understudy (BLEU), Recall-Oriented Understudy for Gisting Evaluation–Longest Common Subsequence (ROUGE-L), BERTScore, and Translation Edit Rate (TER), complemented by redundancy metrics and medical term variation analyses.

**Results:**

LLM-generated notes were significantly longer, more repetitive, and lexically less diverse than human-authored notes. TER-based clustering revealed a uniform, template-like writing style in LLMs, diverging from the flexible, context-sensitive style of physicians. Topic modeling suggested that LLM-generated notes tended to rely on more abstract and generalized expressions, with less variation in the distribution and emphasis of documented clinical information.

**Conclusions:**

LLMs can mimic surface-level stylistic features but fall short in reproducing nuanced, context-dependent, diagnostically relevant content typical of expert clinical documentation. Future clinical use will require careful prompt design or fine-tuning to ensure narrative depth, lexical diversity, and clinical relevance.

## Introduction

Large language models (LLMs) are increasingly being explored in health care for automated documentation, summarization, and decision support [1-7]. Prior work suggests that LLM-generated discharge summaries and notes may achieve acceptable clarity and coverage yet may also omit clinically important details and include redundancy or hallucinations [3,8-14]. The output style varies widely across models and prompt designs [10,15], raising concerns about stability and reproducibility.

Compared with clinical notes written by human physicians, there has been little systematic investigation into the extent to which differences in style and content exist or whether specialty-specific stylistic distinctions are reflected in LLM-generated notes. Although real clinical documentation shows clear stylistic differences across specialties [16-20], it is unclear whether LLMs are capable of reproducing such variations.

In psychiatry in particular, clinical notes are primarily structured around patients’ subjective reports and clinicians’ interpretations, meaning that differences in style and perspective carry direct clinical implications [21]. Psychiatric notes typically integrate not only descriptions of symptoms but also clinical reasoning and risk assessment [22]. Whether an LLM can accurately reproduce such documentation styles directly affects treatment planning, information sharing within the care team, the quality of medical education, and ultimately patient safety [11]. Conversely, if an LLM fails to faithfully imitate the style of psychiatrists, serious clinical risks may arise, including (1) misrepresentation or omission of risk-related information such as suicidal ideation or hallucinations and delusions, which could compromise clinical judgment and patient safety; (2) misunderstandings in multidisciplinary communication, which could reduce the quality of team-based care; and (3) adoption of inappropriate documentation styles by learners, resulting in educational disadvantages [23]. Therefore, verifying LLM-generated clinical notes is not merely a technical comparison but also meaningful from the perspectives of real-world clinical practice, patient safety, and education.

To facilitate a systematic comparison, this study evaluated clinical notes along several analytic dimensions. Specifically, we examined syntactic characteristics, lexical diversity, patterns of redundancy, and topic structure using multiple quantitative natural language processing (NLP)–based metrics. These dimensions were selected to capture complementary aspects of documentation style without presupposing normative judgments about clinical quality.

Against this background, this study aimed to clarify the differences in documentation styles between physicians and LLMs when describing identical psychiatric cases. To this end, we created standardized case scenarios using LLMs and adopted a comparative analytic approach to evaluate initial outpatient notes written by both physicians and LLMs. Through this analysis, we sought to reveal specialty- and generator-specific characteristics and clinical foci, thereby contributing foundational knowledge for future AI-assisted clinical documentation, NLP applications, and educational use.

Therefore, the aim of this study was not to evaluate the maximal performance of clinically fine-tuned language models but rather to examine the extent to which currently available general-purpose LLMs can acquire and reproduce psychiatric documentation styles without additional domain-specific training. This design choice was motivated by our interest in examining how much clinical documentation style can be elicited from general-purpose LLMs through simple role-based prompting alone, without relying on domain-specific fine-tuning.

## Methods

### Language of Materials and Analysis

All study materials and outputs were handled exclusively in Japanese. The standardized case scenarios were prepared in Japanese and presented to clinicians in Japanese. All LLMs were prompted in Japanese and produced Japanese outputs, and the clinician-authored free-text notes were analyzed in their original Japanese form. No translation to or from English was performed at any stage of model prompting, clinician instruction, or quantitative analysis. For readability and accessibility for an international audience, English translations of representative case scenarios, prompts, and example clinical notes are provided in Multimedia Appendix 1; these are for reference purposes only and were not used in any stage of the study.

### Study Design and Materials

First, we constructed two psychiatric outpatient scenarios (depression and schizophrenia) in standardized Japanese, based on items from the Japanese National Medical Licensing Examination (111th B-47 and 112th D-35). These 2 conditions were selected as diagnostically distinct yet prototypical psychiatric presentations, allowing for controlled comparison across specialties and models. The scenarios were intentionally designed to be relatively simple and to limit comorbidities, in order to minimize the confounding effects of case complexity and to focus the analysis on documentation style rather than clinical content heterogeneity. Drafts were generated with assistance from GPT-4o (OpenAI) and subsequently standardized and verified by 2 physicians. During this process, the physicians examined the content for clinical plausibility, internal consistency, and unnatural phrasing that could reflect model-specific artifacts. These scenarios were not intended to represent clinical ground truth or a gold standard but rather to serve as standardized, privacy-preserving reference cases for controlled comparisons of documentation styles. Next, psychiatrists and internists were asked to write free-text initial consultation notes in Japanese for each scenario. They were explicitly informed that the scenarios had been generated with the assistance of an LLM and were instructed to treat them as real clinical cases, freely modifying, supplementing, or correcting the content as needed, including addressing missing or unrealistic elements. In addition, 4 LLMs were given the same case scenarios and instructed to generate clinical notes, using the prompt shown in Textbox 1. The only difference from the physician instructions was the role designation at the beginning; otherwise, the text was identical.

**Figure 1.**
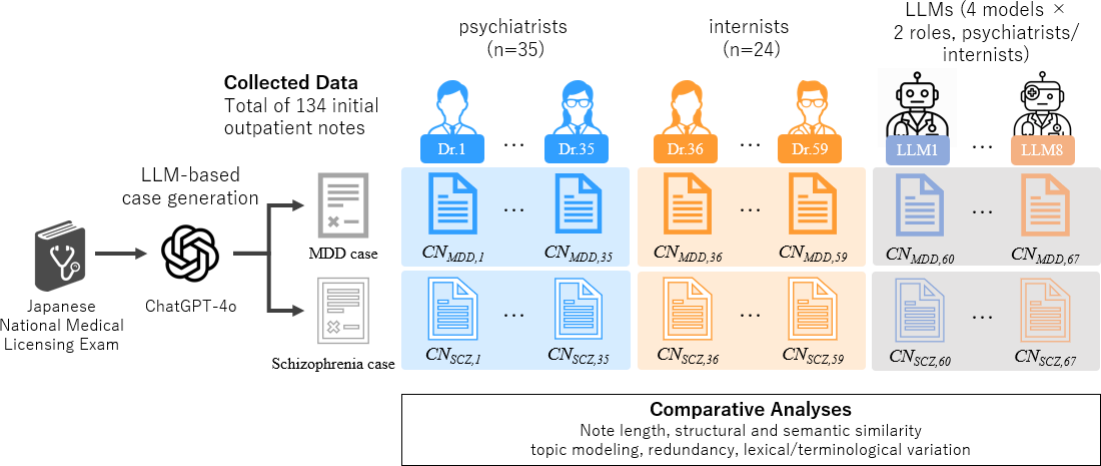
Overall study workflow: from case creation to note collection and analytic procedures. LLM, large language model; MDD, major depressive disorder; CN, clinical note.

The overall study workflow is shown in Figure 1.

Textbox 1. **Clinical note generation prompt (English translation of the original Japanese prompt)**

You are a psychiatrist (or an internist). Based on the scenario, please create an initial psychiatric outpatient note (summary). The format can follow the conventions of your institution. If there are missing elements (eg, differential diagnoses, questions needed to guide treatment) or unnatural aspects, please supplement or revise the content based on your clinical experience and reflect this in the note.

### Participants and Models

Physicians were recruited via the medical research platform m3.com. A total of 35 practicing psychiatrists and 24 internists participated in the study. The LLMs used in this study included widely available cloud-based models (GPT-4o and Claude Sonnet 4 [Anthropic]), as well as Japan-compatible local models suitable for medical applications (ELYZA/LLaMA3-8B [ELYZA, Inc] and DeepSeek-R1/Qwen-14B [CyberAgent, Inc]). This selection allowed comparisons from both performance and practical applicability perspectives. All models were run with their publicly available default parameters. The local models were executed via LM Studio (version 0.3.22). Generation parameters were user controllable and were fixed at temperature=0.8, top_p=.95, context length=4096, and repeat penalty=1.1 for all experiments. Text generation was performed using standard stochastic sampling, without greedy decoding or beam search. The cloud models were accessed through their graphical user interface versions, which do not permit user modification of generation parameters, and thus were used with default configurations.

Each physician and each LLM produced notes for both the depression and schizophrenia cases. In total, 70 psychiatrist notes, 48 internist notes, 4 LLM psychiatry-style notes, and 4 LLM internal medicine–style notes were collected. Excerpts from psychiatrist and internist notes for the depression and schizophrenia cases are presented in Table 1.

**Table 1.** Representative excerpts from physician-authored initial notes.

Case	Physician attributes	Note excerpt
Depression	In their 50s; board-certified psychiatrist	HPI^a^: after promotion to section chief, depressive mood worsened with increased work errors, dizziness, fatigue, insomnia, and appetite loss; an occupational physician recommended a psychiatry referral; mild suicidal ideation without a specific plan Assessment and plan: suspect major depressive disorder; provide supportive psychotherapy; consider an antidepressant; discuss workload adjustment including possible sick leave
Depression	In their 50s; general internist	S^b^: insomnia, fatigue, appetite loss, suicidal ideation O: appears depressed A: stress-related depressive state after promotion is suspected. P: SSRI^c^ + environmental adjustments
Schizophrenia	Man in his 20s; board-certified psychiatrist	HPI: 1 week of insomnia and odd behaviors after a graduate-school entrance exam; found pacing and muttering for about 3 hours, poor eye contact, intermittently responding to unseen stimuli Workup: laboratory tests, brain MRI^d^, and EEG^e^ unremarkable Assessment: schizophrenia spectrum disorder versus unspecified psychotic disorder Plan: supportive psychotherapy, consider antipsychotic, psychoeducation for family, follow-up and possible psychological testing
Schizophrenia	In their 50s; general internist	S: hallucinations, delusions O: communication is difficult A: schizophrenia suspected P: atypical antipsychotic + psychosocial support

a HPI: history of present illness.

b SOAP: subjective, objective, assessment, and plan.

c SSRI: selective serotonin reuptake inhibitor.

d MRI: magnetic resonance imaging.

e EEG: electroencephalogram.

### Conceptualization of Documentation Characteristics in Psychiatric Notes

Clinical reasoning in psychiatry extends beyond factual reporting and encompasses hypothesis generation, differential diagnosis, structured risk formulation, and justification of clinical decisions [24-26].

However, written clinical notes do not directly represent these internal cognitive processes; rather, they constitute narrative documents through which clinicians organize, emphasize, and integrate patient information. Accordingly, this study did not attempt to measure clinical reasoning itself. Instead, we focused on observable linguistic and structural characteristics of documentation, operationalized using a set of complementary NLP metrics. Structural edit patterns, topic dispersion, lexical specificity, and redundancy were treated as descriptive indicators of how information is distributed, repeated, and integrated within clinical notes. For example, topic dispersion and lexical specificity were interpreted as reflecting the breadth and focus of documented clinical concerns, while structural edit patterns and redundancy patterns captured the extent to which information is synthesized versus descriptively repeated.

This documentation-centered operationalization enabled a systematic comparison of note-writing styles across human- and LLM-authored records, without assuming that these linguistic features constitute validated proxies for underlying reasoning quality or internal cognitive processes.

Importantly, although certain analyses (eg, topic modeling and term frequency-inverse document frequency [TF-IDF] of diagnostic terms) involved lexical and thematic content, they were interpreted in this study as indicators of how information was distributed, emphasized, and structured within the document rather than as evaluations of diagnostic accuracy or substantive clinical correctness. Accordingly, our focus remained on observable documentation patterns rather than on the validity or quality of clinical reasoning itself.

### Analytic Overview

We conducted multidimensional analyses of length, lexicon, syntax, semantics, topics, redundancy, and term variation [14,27]. This multimetric analytic framework enabled a quantitative comparison of documentation styles while maintaining clinical interpretability, helping to distinguish surface-level textual similarity from patterns that reflect how clinically relevant information is organized and emphasized in psychiatric notes. The clinical interpretation of each NLP metric used in this study is summarized in Table 2.

**Table 2.** Clinical interpretation of natural language processing metrics in psychiatric clinical notes.

Metric	Captured textual dimension	Intended clinical interpretation
TER^a^	Structural divergence between texts	Degree of structural divergence between notes; reflects how much rewriting is required to transform one note into another
BLEU^b^/ROUGE-L^c^	Surface-level n-gram overlap	Similarity of phrasing and boilerplate expressions
BERTScore	Semantic similarity	Preservation of overall clinical meaning independent of wording
TF-IDF^d^	Lexical specificity	Use of diagnostically and clinically salient terminology
Topic modeling (LDA^e^)	Thematic structure	Breadth and focus of clinical concerns addressed in the note
Redundancy metrics	Information compression versus repetition	Balance between concise synthesis and template-driven verbosity
Medical term variation	Terminological consistency and variability	Variability in diagnostic and risk-related terminology, reflecting diagnostic precision, risk framing, and alignment with psychiatric conventions

a TER: Translation Edit Rate.

b BLEU: Bilingual Evaluation Understudy.

c ROUGE-L: Recall-Oriented Understudy for Gisting Evaluation–Longest Common Subsequence.

d TF-IDF: term frequency-inverse document frequency.

e LDA: latent Dirichlet allocation.

With respect to LLM-generated notes, to reduce model-specific idiosyncrasies and focus on role-level tendencies, we aggregated the results of all 4 LLMs within each prompted role.

### Descriptive Statistics

We computed note length statistics such as mean, median, and variance by specialty and generator, and we tested group differences using *t* tests and ANOVA for each scenario. Because the number of LLM-generated notes per model was small (n=2 per condition), we used nonparametric tests (Mann-Whitney U test) and reported effect sizes using Cliff delta. These analyses were intended as descriptive sensitivity analyses rather than definitive inferential tests.

### Syntactic-Semantic Similarity

To compare the syntactic structures and semantic features of clinical notes, we computed pairwise sentence-level similarity across notes and visualized the results using hierarchical clustering. We used four metrics commonly used in NLP for sentence similarity evaluation:

- Translation Edit Rate (TER): TER evaluates structural similarity based on the number of editing operations (insertion, deletion, substitution, reordering) required to transform one sentence into another [28]. It is defined as follows (lower values indicate higher structural similarity to the reference sentence):

$$TER=\frac{\text{Number of edits}}{\text{Number of words in reference sentence}}$$

- Bilingual Evaluation Understudy (BLEU): BLEU is a machine translation evaluation metric based on n-gram overlaps and reflects the local syntactic consistency of sentences [29]. Higher values indicate a higher syntactic similarity to the reference.
- Recall-Oriented Understudy for Gisting Evaluation–Longest Common Subsequence (ROUGE-L): ROUGE-L measures syntactic overlap between 2 sentences based on the length of the longest common subsequence [30]. Higher values indicate a higher syntactic similarity.
- BERTScore: It assesses semantic similarity by comparing token embeddings generated by a pretrained bidirectional encoder representations from transformers (BERT) model [31]. Higher values indicate higher semantic similarity. BERTScore was computed using the bert-score package (version 0.3.13;[32]), with the language set to Japanese (lang=“ja”) and the pretrained model set to xlm-roberta-large (model_type=“xlm-roberta-large”). We used the *F*_1_-score for analysis, with batch_size=32 and computation performed on the specified device (central processing unit/graphics processing unit).

Using these metrics, we calculated pairwise similarity between notes. For each pair of notes denoted as $CN_{A}$ and $CN_{B}$, when the metric produced directional scores such as TER and BLEU, we averaged the scores in both directions ($CN_{A}\to CN_{B}$ and $CN_{B}\to CN_{A}$) to determine the similarity. Accordingly, we interpreted TER as a measure of overall structural divergence between 2 notes rather than as a unidirectional indicator of overdocumentation or underdocumentation. For each metric, a distance matrix was constructed from the similarity scores, and hierarchical clustering using Ward’s [33] method was applied. This enabled the grouping of notes on the basis of their syntactic and semantic tendencies. We first normalized all texts using Unicode normalization (NFKC), standardized white space, and removed Japanese punctuation marks. We then tokenized the Japanese text using MeCab (version 0.996; developed by Taku Kudo) to obtain white space–separated tokens for the metric computations. Although BLEU and ROUGE-L were originally developed for machine translation and summarization tasks, respectively, in this study, they were used in a descriptive manner to quantify the surface-level n-gram overlap and reuse of stereotyped phrasing between texts rather than to assess translation or summarization quality. These metrics have known limitations in evaluating free-text clinical documentation, as they emphasize surface-level n-gram overlap and may not fully capture higher-order discourse structure or clinically meaningful nuance. Therefore, in this study, they were used strictly as descriptive indicators rather than as comprehensive quality measures.

### Lexical Analysis

We evaluated the lexical usage patterns across the entire set of notes using TF-IDF [34]. This is a weighted measure calculated as the product of a term’s frequency in a document and the inverse document frequency, which reflects how specific the term is across the full set of documents:


$$\text{TF-IDF}(t,d)=\text{TF}(t,d)\times log\left(\frac{N}{\text{DF}(t)}\right)$$

Here, t denotes a term, d denotes a document, N is the total number of documents, TF(t,d) denotes the number of times the term *t* appears in document *d*, and DF(t) is the number of documents containing the term t. On the basis of this definition, the Japanese texts were tokenized using SudachiPy (version 0.6.10; Works Applications Co, Ltd), with rule-based merging of multiword medical expressions and removal of non-informative tokens. TF-IDF features were then computed using scikit-learn (version 1.6.1) [35]. To ensure the comparability of TF-IDF values across groups, we concatenated all documents from the 4 groups and fitted a single TF-IDF vectorizer on the combined corpus so that the vocabulary and IDF weights were shared across all groups. We then computed the mean TF-IDF score for each term within each group and reported the top-ranked terms accordingly.

### Topic Modeling

We applied latent Dirichlet allocation (LDA) [36] to visualize thematic tendencies, normalizing the topic scores by group size.

### Medical Term Variation

Using the standardized medical dictionary JMED-DICT [37], we extracted medical terms such as disease and symptom names from the notes and analyzed the variation in their lexical forms by specialty and by author type, comparing human physicians with LLMs. For this analysis, we focused specifically on lexical variants related to “depressive state,” including “depressed mood,” “loss of motivation,” “depression,” “depressive state,” and “major depressive disorder.” The diversity of expressions for medical terms was quantified using lexical entropy, also known as Shannon entropy, which measures the variation in lexical forms used for the same medical concept. A larger value indicates greater diversity of expressions. Lexical entropy is defined as


$$H=-\sum_{i=1}^{n}p_{i}{log}_{2}p_{i}$$

where $p_{i}$ represents the probability of occurrence of lexical form i.

### Redundancy

To quantitatively compare redundancy in clinical notes across specialties and author types, we evaluated repetition and lexical diversity biases in the text using the following 6 metrics; these measures are effective for capturing structural repetition and lexical limitation or excess:

- Number of sentences: The text was segmented by periods or line breaks into sentences, and the total number of sentences was counted.
- Lexical diversity (type-token ratio; TTR): The proportion of unique words to the total number of words was calculated. Lower values indicate more lexical repetition and thus higher redundancy.

$$TTR=\frac{\text{Number of unique words}}{\text{Total number of words}}$$

- Inter-sentence similarity: The semantic similarity between sentence vectors within the same text was measured. Each sentence was vectorized using TF-IDF, and redundancy was evaluated with the average cosine distance between sentences.

Redundancy=1-Average cosine distance

- Compression ratio: The text was compressed using the DEFLATE algorithm, which combines LZ77 and Huffman coding, and the ratio of compressed to original size was calculated. Redundant text is compressed more efficiently, resulting in a lower compression ratio.

$$\text{Compression ratio}=\frac{\text{Compressed size}}{\text{Original size}}$$

- Unique words per sentence: The average number of unique words per sentence was calculated to evaluate the lexical density. Lower values indicate that sentences are composed of limited vocabulary and thus exhibit higher redundancy.
- Bigram duplication rate: The proportion of repeated adjacent 2-word sequences (bigrams) in the text. Higher values indicate stronger tendencies toward lexical repetition.

$$\text{Duplication rate}=1-\frac{\text{Number of unique bigrams}}{\text{Total number of bigrams}}$$

These metrics allow an objective evaluation of redundant vocabulary and sentence structures in clinical notes and provide insights into stylistic differences across specialties and author types. From a clinical documentation perspective, these redundancy patterns describe how information is distributed and repeated within notes, without directly assessing informational value or clinical adequacy.

### Ethical Considerations

This study did not involve real patient data. All clinical cases used in the analysis were fully simulated scenarios created for research purposes and did not contain any identifiable personal information.

The physician-authored clinical notes were collected from licensed medical doctors who voluntarily participated in the study through an online medical research platform (m3.com). Informed consent was obtained from all participating physicians prior to data collection, and participants were informed of their right to withdraw or opt out of the study at any time without any consequences. No patient-related information was included, and all physician responses were anonymized before analysis.

Because the study involved only simulated clinical cases and anonymous textual data provided by consenting medical professionals, it did not constitute research involving human subjects under institutional regulations. According to Article 4 of the “Regulations on Life Science and Medical Research Involving Human Subjects” of Nara Institute of Science and Technology (NAIST) [38], studies that do not involve human participants, biological samples, or identifiable personal information fall outside the scope of human subject research and are not subject to institutional review board (IRB) review. As this study used fully synthetic case scenarios and did not include any patient data or identifiable personal information, formal IRB approval was not required.

## Results

### Descriptive Statistics

This subsection summarizes the quantitative differences in note length across specialties and generating agents, providing a baseline characterization of documentation volume before examining the structural and semantic features.

**Figure 2.**
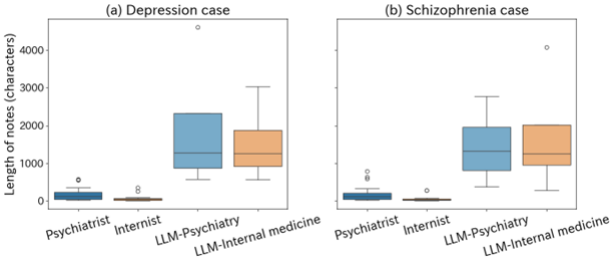
Note length distributions by specialty and generator for depression (A) and schizophrenia (B). LLM, large language model.

**Figure 3.**
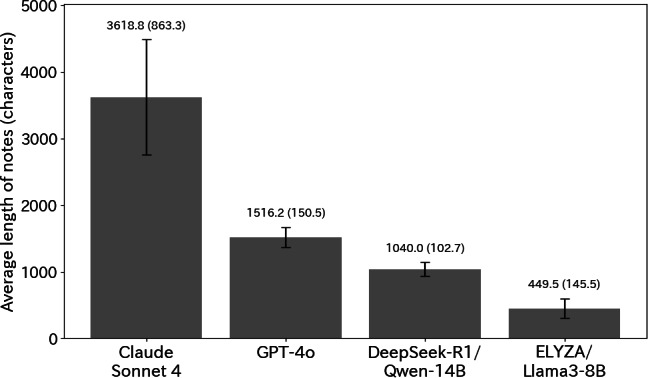
Mean (SD) note length by large language model.

For both the depression and schizophrenia cases, we compared the length of notes between groups. Figure 2 visualizes the full distribution and dispersion of note lengths, highlighting the variability and overlap across specialties and generating agents that are not fully captured by mean values alone. We calculated the mean, median, and variance and assessed the statistical significance of group differences using ANOVA 2-tailed *t* tests. As a result, clear differences in note length were observed across groups for both depression (*F_3,130_*=27.08, *P*<.001) and schizophrenia cases (*F_3,130_*=25.73, *P*<.001). In contrast, no significant differences were found between LLM-psychiatry and LLM-internal medicine notes for either depression (*t_6_*=0.38, *P*=0.72) or schizophrenia (*t_6_*=–0.28, *P*=0.79). Further comparisons of note length across individual LLM models (Figure 3) showed that Claude Sonnet 4 produced the longest outputs, followed by GPT-4o, DeepSeek-R1/Qwen-14B, and ELYZA/LLaMA3-8B in decreasing order of mean length. Figure 3 enables a direct visual comparison of length variability across individual LLMs, complementing the aggregated analyses presented in Figure 2. Claude Sonnet 4 also exhibited a larger SD than the other models, suggesting greater variability in its outputs. These findings indicate that note style and information density varied across different LLM models.

To examine whether the observed differences in note length were driven by a specific LLM, we conducted model-wise comparisons between clinician-authored notes and notes generated by each LLM independently using the Mann-Whitney U test. Effect sizes were quantified using Cliff delta. Across all evaluated models, LLM-generated notes were consistently longer than clinician-authored notes for both depression and schizophrenia cases (all Cliff *δ*≥0.86). These differences remained consistent after false discovery rate correction, and effect sizes were large across all evaluated models. These findings indicate that the aggregated length differences observed in Figures2  3 were not driven solely by a single model (eg, Claude Sonnet 4) but reflected a consistent pattern across all tested LLMs.

Given the small number of LLM-generated notes per condition (n=4 per role), these statistical comparisons should be interpreted as exploratory and primarily descriptive rather than confirmatory population-level inferences. Accordingly, the reported *P* values and effect sizes are presented to characterize the distributional tendencies within the sampled texts rather than to support definitive generalizable claims.

### Syntactic and Semantic Similarity

This subsection describes how clinical notes clustered according to syntactic and semantic similarity, to clarify whether differences in documentation style across specialties and generators were reflected in structural edit patterns and surface-level phrasing.

**Figure 4.**
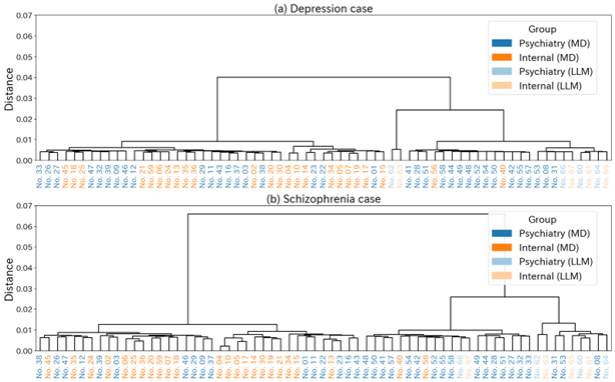
Hierarchical clustering of clinical notes based on syntactic similarity (Translation Edit Rate) for the depression case (A) and schizophrenia case (B). MD, human physician; LLM, large language model.

In the clustering based on TER, the differences in charting style across specialties and generating agents were clearly reflected (Figure 4). This figure provides a structural, distance-based visualization of documentation styles, revealing systematic separation patterns that are not apparent from pairwise similarity scores alone. In both the depression and schizophrenia cases, psychiatrists’ notes and internists’ notes written by human physicians formed distinct clusters, and this difference in syntactic features consistently appeared regardless of the case. Of particular note is that the notes generated by LLMs diverged substantially from those written by human physicians, with LLM-psychiatry and LLM-internal medicine each forming their own independent clusters. This suggests a tendency for LLM-generated notes to exhibit generation styles and sentence structures that differ from those of human physicians’ records. In contrast, hierarchical clustering based on surface-level n-gram similarity using BLEU or ROUGE-L did not produce a clear separation between clinician-authored and LLM-generated notes (Appendix D—Figures S1 and S2 in Multimedia Appendix 1), suggesting that these metrics were less sensitive to structural differences in charting style. Clustering based on BERTScore, which reflects proximity in contextual embedding space, resulted in substantial cross-specialty and cross-agent mixing (Appendix D—Figure S3 in Multimedia Appendix 1). This finding indicates that shared diagnostic terminology and case descriptions may dominate embedding-based similarity and should be interpreted as reflecting relative semantic relatedness rather than similarity in clinical reasoning or documentation intent.

The factors behind the clear separation achieved by TER include its high sensitivity to syntactic changes such as word order, insertion, and deletion, in addition to the influence of differences in text length. In this study, we also independently calculated the relative numbers of insertions, deletions, and substitutions, normalized by the reference sentence length, and compared them across specialties and generating agents. Shift operations were not considered due to computational cost. The comparison results are shown in Table 3.

**Table 3.** Average relative values of each editing operation (per sentence length) across specialties and generating agents.

Comparison groups	Insertion	Deletion	Substitution
Psychiatrists-Psychiatrists	2.41	0.13	0.71
Internists-Internists	2.20	0.10	0.76
Psychiatrists-Internists	3.89	0.12	0.70
Psychiatrists-LLM^a^ Psychiatry	20.81	0.00	0.63
Psychiatrists-LLM Internal Medicine	15.05	0.00	0.64
Internists-LLM Psychiatry	49.56	0.00	0.50
Internists-LLM Internal Medicine	36.20	0.00	0.49
LLM Psychiatry-LLM Psychiatry	0.42	0.46	0.42
LLM Psychiatry-LLM Internal Medicine	0.25	0.37	0.47
LLM Internal Medicine-LLM Internal Medicine	0.17	0.38	0.50

a LLM: large language model.

As a result, among human physicians (psychiatry-psychiatry, internal medicine-internal medicine), editing operations were relatively few, indicating stable charting styles. In contrast, between human physicians and LLMs, the average relative numbers of insertions were remarkably high, with that for internal medicine physicians and LLM-psychiatry reaching an outstanding 49.56. This suggests that LLMs tend to include more information than human physicians. The frequencies of deletions and substitutions were relatively stable, indicating that the differences in information presence (particularly the number of insertions) contributed most strongly to increasing the structural distance measured by TER. Furthermore, in comparisons among LLMs, the relative numbers of insertions were extremely low (around 0.2‐0.4), indicating similarity in text length, whereas the frequencies of deletions and substitutions were somewhat higher, reflecting stylistic differences in syntax and word order.

Taken together, syntactic distance based on TER served as a descriptive indicator of differences in charting styles across specialties and generating agents, highlighting structural characteristics that differed between LLM-generated and human-authored notes. These results suggest that TER is a useful descriptive metric for characterizing chart structure in medical notes, particularly when interpreted alongside complementary lexical and semantic analyses.

Because the number of LLM-generated notes per condition was limited, these clustering patterns should be interpreted as descriptive structural tendencies rather than statistically validated group separations. The results therefore characterize relative distributional patterns within the present sample rather than population-level distinctions.

### Lexical Analysis

#### Analysis of Differences in Vocabulary Usage

This subsection outlines the differences in vocabulary usage across specialties and generating agents using TF-IDF, highlighting how clinically salient terms are distributed and emphasized in each group.

Using the TF-IDF scores, we analyzed the differences in vocabulary usage across specialties and generating agents, comparing human physicians and LLMs, as shown in Table 4 and Multimedia Appendix 1 (Appendix D—Table S1).

**Table 4.** Top TF-IDF^a^–weighted terms by group for the depression case.

Group	TF-IDF
Psychiatrists	
Decrease	0.083
Depression	0.074
None	0.064
Initiation	0.063
Symptoms	0.055
Suicidal ideation	0.055
Mood	0.053
Therapy	0.054
Depression (disease)	0.053
Mental	0.048
Internists	
Depression	0.099
Condition	0.099
Suicidal ideation	0.095
SSRI	0.075
Insomnia	0.058
Male	0.054
Mild	0.056
Depression (term)	0.064
Initiation	0.058
45	0.070
LLM^b^ psychiatry	
Family	0.138
Mental	0.112
Symptoms	0.107
Patient	0.102
Evaluation	0.084
Decrease	0.085
Treatment	0.081
Disorder	0.079
Function	0.077
Tendency	0.076
LLM internal	
Family	0.168
Patient	0.146
Symptoms	0.142
Necessary	0.120
Diagnosis	0.105
None	0.112
Effect	0.105
Function	0.100
Test	0.099
Mental	0.099

a TF-IDF: term frequency-inverse document frequency.

b LLM: large language model.

#### Depression Case

In the notes written by psychiatrists, terms such as “decrease,” “depression,” “mood,” and “suicidal ideation” appeared with high scores, reflecting emotional changes and psychiatric symptoms. In addition, words such as “initiation,” “therapy,” and “mental” were also included, focusing on treatment initiation and psychological aspects. In the notes written by internists, terms such as “condition,” “SSRI,” “insomnia,” and “mild” were more common, reflecting standardized vocabulary related to clinical findings and treatment content. In LLM-generated notes, the psychiatry-style outputs contained many abstract terms such as “family,” “mental,” “symptoms,” and “evaluation.” While structurally mimicking psychiatric-style records, they lacked clinical specificity. Similarly, in the internal medicine–style outputs, many generic terms such as “family,” “patient,” “necessary,” and “symptoms” were observed, which differed from the vocabulary actually used by human internists.

#### Schizophrenia Case

In the notes written by psychiatrists, the high-scoring and frequently occurring words included “schizophrenia,” “mental,” “auditory hallucination,” “risperidone,” and “behavior,” reflecting diagnostic reasoning, clinical observations, and medication names. By contrast, internists often used standardized terms such as “schizophrenia,” “suspected,” “typical,” “oral medication,” and “abnormal,” focusing primarily on diagnostic labels and treatment actions. In the LLM psychiatry–style notes, more general and abstract words such as “family,” “mental,” “symptoms,” and “university” appeared, while symptom descriptions and drug names, which were common in psychiatrist-authored notes, were scarce. A similar vocabulary pattern was observed in the LLM internal medicine–style outputs.

#### Overall Considerations

In human-authored notes, psychiatrists tended to use context-dependent and multidimensional vocabulary to describe patients’ subjective experiences and the treatment process, whereas internists emphasized objective findings and standardized judgments. LLMs partially mimicked these stylistic features, but their outputs tended to converge on frequently used and generic words such as “family” and “mental.” This suggests that LLM-generated notes were prone to rely on standardized and generalized vocabulary. TF-IDF–based lexical analysis was shown to be an effective method for visualizing and quantifying such stylistic differences in clinical documentation.

### Topic Analysis

This subsection outlines the thematic tendencies in clinical notes using topic modeling, illustrating how different generators and specialties allocate attention across abstract descriptions, diagnostic reasoning, and observed findings.

**Figure 5.**
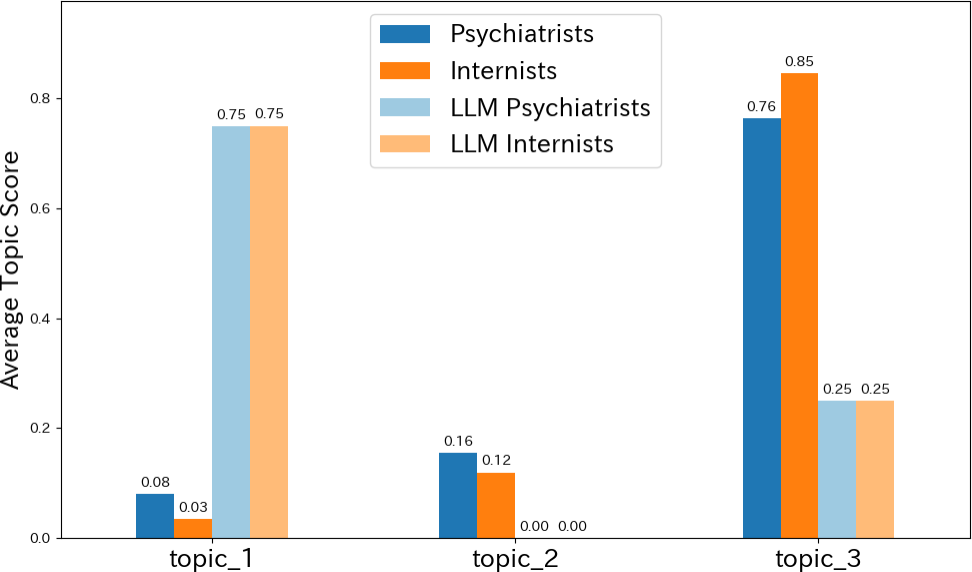
Topic distributions by specialty and generating agent (depression and schizophrenia cases) based on latent Dirichlet allocation. LLM, large language model.

We conducted a topic analysis using LDA on all clinical notes generated by psychiatrists, internists, and LLMs for the 2 cases of depression and schizophrenia, identifying 3 major topics. As shown in Figure 5, the distribution of average topic scores clearly reflected differences across generating agents and medical specialties. Figure 5 highlights how thematic emphasis differs across groups at an aggregate level, offering an interpretable overview that complements the term-level results reported in Table 5. The representative words and summaries of each topic are presented in Table 5.

**Table 5.** Topics and associated words.

Topic	Associated words (top contributors)
Topic 1 (abstract and comprehensive description)	Mental, family, symptoms, none, disorder, treatment, evaluation, diagnosis, behavior, physical
Topic 2 (diagnostic reasoning and treatment planning)	Mental, condition, treatment, future, therapy, depression, course, suicidal ideation, medication, hallucination
Topic 3 (observed findings and stereotyped records)	None, insomnia, initiation, schizophrenia, symptoms, depression, suicidal ideation, condition, male, abnormal

Since topic scores depend on the number of words and notes, we calculated the normalized average scores for each specialty and generating agent for comparison (Figure 5). The results showed that psychiatrists had high scores in topic 2 (diagnostic reasoning and treatment planning) and topic 3 (observed findings and stereotyped records), suggesting that their documentation was based on both symptom observation and clinical reasoning. In contrast, internists exhibited an extreme bias toward topic 3, focusing primarily on stereotyped documentation of observed findings, with limited engagement in psychiatric symptoms or treatment planning.

Meanwhile, LLM-generated notes (for both psychiatry and internal medicine) were markedly biased toward topic 1 (abstract and comprehensive description), converging on template-like descriptions centered on frequent terms such as “family,” “mental,” and “none.” These results suggest a descriptive pattern in which LLM-generated notes tended to converge on abstract and generalized expressions, whereas human physicians’ notes more frequently reflected context-dependent descriptions and discipline-specific emphases.

### Expression Style and Diversity of Medical Terms

This subsection focuses on the variation in lexical expressions for equivalent medical concepts, using lexical entropy to compare terminological diversity across specialties and generators.

As a preliminary examination of lexical diversity, this study focused on the variation of expressions related to “depressive state” and compared the differences in word forms across medical departments and generation sources. Using a normalized medical dictionary, we extracted word forms judged to be semantically equivalent to “depressive state” and calculated the lexical entropy based on the frequency of each word form. As a result, the medical records written by psychiatrists exhibited the greatest diversity of expressions, with a lexical entropy of **3.206**. Specifically, a variety of terms such as “depression,” “depressed mood,” “depressive mood,” “depressive state,” “depressive symptoms,” and “melancholy” were observed. In contrast, internists used only limited and formulaic expressions such as “depressive state,” “depression,” or “depressive condition,” yielding a lower entropy of **2.000**.

In the LLM-generated records, the psychiatric style showed an entropy of **2.653**, while the internal medicine style showed an entropy of **2.322**. These values indicate an intermediate level of lexical diversity compared with human physicians. Nevertheless, the range of lexical choices remained restricted. These results suggest that in psychiatry, even for the same medical concept, physicians tend to flexibly choose expressions according to the patient’s wording and context. Although the outputs of LLMs partially imitate this tendency, they still show a bias toward template-like usage.

### Redundancy Analysis

This subsection focuses on redundancy and information density in clinical notes using multiple complementary metrics, clarifying whether increased length corresponds to effective information integration or repetitive structure.

**Figure 6.**
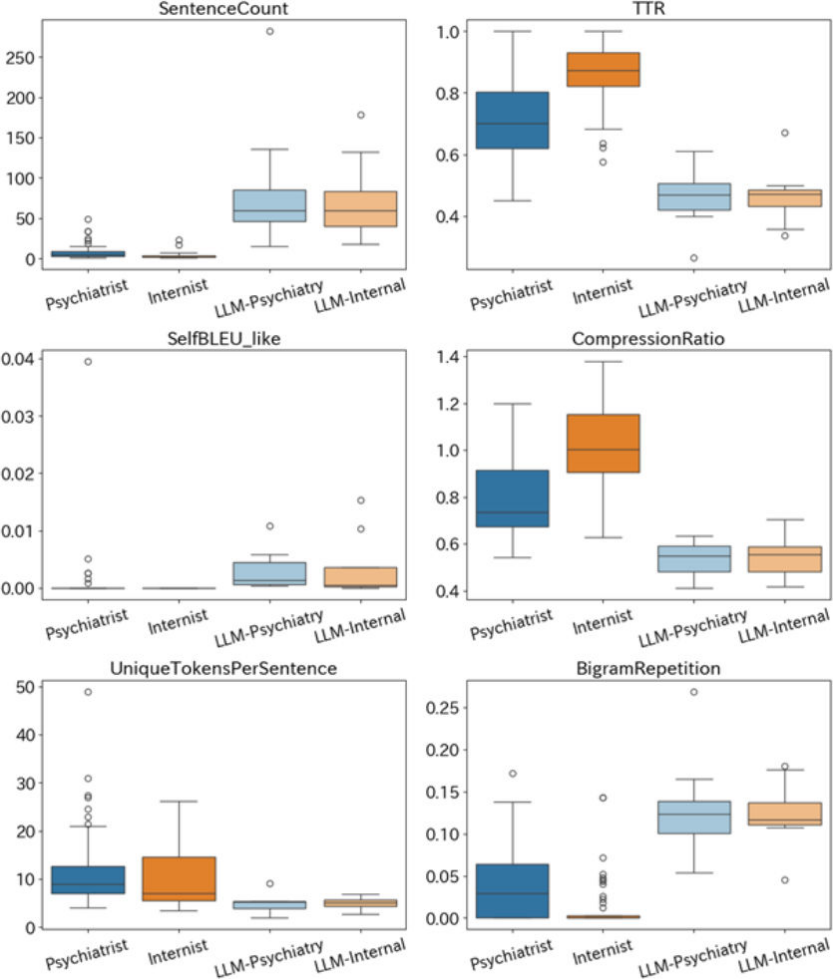
Redundancy and efficiency indicators across groups, showing that large language models (LLMs) exhibit greater repetition and lower lexical diversity. LLM-Internal, LLM-internal medicine.

To quantitatively evaluate the redundancy, information density, and lexical diversity in medical records, we analyzed each group’s documentation using 6 linguistic indicators (Figure 6). This figure integrates multiple redundancy-related metrics into a single visual summary, facilitating a cross-metric comparison of repetition and information density that would be difficult to infer from individual statistics alone. The records generated by LLMs exhibited markedly different characteristics from those written by human physicians across all indicators. In particular, the number of sentences was highest in both psychiatric and internal medicine styles, suggesting a tendency for LLM-generated records to become lengthy. In contrast, the lexical diversity and number of unique words per sentence were notably low, strongly indicating structural repetition and lexical redundancy. For redundancy-related indicators such as inter-sentence similarity, bigram repetition rate, and compression ratio, the LLM groups also showed higher values (or lower compression ratios). This suggests that a reliance on specific template structures and frequent reuse of expressions may limit the effective density of information.

On the other hand, human physicians’ records—especially those generated by psychiatrists—demonstrated higher lexical diversity and more unique words per sentence, providing dense, multifaceted information despite being relatively concise. Records written by internists were generally shorter and relied more on standardized phrasing; however, unlike LLM outputs, they displayed lower repetitiveness and thus higher informational efficiency. These findings suggest that while LLM-generated medical documents may superficially contain abundant information, they exhibited lower lexical diversity and higher repetition across multiple redundancy-related indicators, compared with human-authored notes. This highlights important areas for improvement in the future application of LLMs to medical documentation.

To assess whether these redundancy-related differences could be explained by sample size imbalance alone, we conducted a bootstrap-based sensitivity analysis in which physician-authored notes were repeatedly downsampled to match the LLM sample size (n=4). Several key redundancy metrics (eg, BigramRepetition and UniqueTokensPerSentence) remained systematically more extreme for LLM-generated notes, often lying outside the 95% range of the bootstrapped distributions of human physician–authored notes. This suggests that these redundancy-related patterns are unlikely to be attributable to small-sample effects alone.

## Discussion

### Significance of Clinical Documentation Style

In this study, we compared the records of psychiatrists, internists, and LLMs (psychiatric and internal medicine styles) for 2 cases of depression and schizophrenia and examined the documentation style from multiple dimensions, including syntax, semantics, vocabulary, topics, redundancy, and lexical diversity. Psychiatric documentation is not merely an administrative record but a core component of clinical communication and continuity of care, as it must capture patients’ subjective experiences, contextual information, and longitudinal illness narratives. Because psychiatric practice is strongly language-centered, it has been regarded as a particularly promising domain for LLM-assisted clinical support [39]. However, recent studies indicate that while LLMs can improve surface-level fluency and efficiency, they often struggle to preserve clinically meaningful nuances, such as temporal relationships and context-dependent interpretation [40]. In sensitive domains such as suicidality and risk assessment, stylistic differences in documentation may influence how information is perceived or interpreted in downstream clinical or educational contexts [41], although the present analyses do not directly evaluate clinical safety or decision-making outcomes. From this perspective, stylistic alignment with human clinicians is not a cosmetic issue but a safety-relevant and epistemically meaningful property of psychiatric documentation. Accordingly, our comparative analysis was intended to examine whether current LLMs approximate deeper, domain-specific documentation practices rather than merely producing plausible-looking clinical text.

### Principal Findings

A particularly striking finding was the substantial stylistic differences observed between human physicians and LLMs. Physicians’ notes were characterized by richer lexical diversity and syntactic flexibility, often reflecting patients’ subjective experiences and contextual backgrounds. By contrast, LLM-generated records displayed limited lexical choices, frequent repetition of similar expressions, and reliance on stereotypical syntactic patterns. Measures such as lexical entropy and redundancy further signified the uniformity of LLM-generated documentation. Lexical and topic analyses further suggested that LLM-generated notes were less likely to include certain linguistic patterns commonly associated with social context and psychological observation in psychiatrist-authored notes. Importantly, these findings should be interpreted as quantitative signals derived from textual features rather than as direct evidence of impaired or absent clinical reasoning.

We also found differences among LLMs in terms of document length and information density. Models such as Claude Sonnet 4, which produce longer outputs, appeared at first glance to provide richer information, but the content often included abstract phrasing and repetition, resulting in lower informational efficiency. On the other hand, models producing shorter records maintained conciseness but risked omitting clinically essential information. Thus, the suitability of LLMs for medical record generation should not be judged merely by their capacity for long text production but rather by their ability to adapt to discipline-specific styles, select clinically meaningful content, and provide concise yet dense documentation.

Another notable finding was that the clear stylistic differences between psychiatric and internal medicine records observed among human physicians were not reproduced in the LLM outputs. Even when explicitly prompted to take the role of a psychiatrist or internist, LLMs tended to converge toward similar, template-like styles, failing to adequately capture the discipline-specific expressions and clinical emphases evident in human-authored notes. While alternative prompting strategies, such as structured note templates or few-shot exemplars, may plausibly influence surface-level characteristics of LLM-generated records, including length, sectioning, and the inclusion of predefined content categories. They are less likely to fundamentally alter the deeper aspects of documentation observed in this study, such as lexical diversity, topic breadth, and the integration of social and psychological context. These higher-order features reflect not only prompt compliance but also the internal representations and training distributions of the models. Accordingly, although prompt engineering may partially mitigate stylistic uniformity, the persistent convergence observed across conditions suggests limitations that cannot be fully addressed through prompting alone. This suggests that merely assigning a role through prompting is insufficient for acquiring domain-specific styles of reasoning and documentation. Future approaches may require domain-adaptive training, fine-tuning, or integration with external knowledge sources to address this limitation. Taken together, these findings indicate that the automatic generation of medical documentation requires more than simply producing natural-sounding sentences. Higher-level capabilities are needed, including contextual understanding, recognition of the documentation purpose, and adaptation to the appropriate documentation style. Future developments in medical NLP should therefore incorporate approaches that engage more directly with the substantive content of records and model the underlying documentation practices.

These findings should therefore be interpreted as a stylistic and structural characterization of documentation patterns rather than an assessment of the clinical adequacy or correctness of the documented content.

### Implications for Clinical Education and Patient Safety

It is important to emphasize that the metrics used in this study are descriptive indicators of textual structure and organization rather than validated measures of clinical quality, safety, or educational adequacy. Accordingly, the following implications should be interpreted as potential considerations rather than direct evaluations of risk. The observed stylistic convergence and increased redundancy in LLM-generated psychiatric notes have important implications for both clinical education and patient safety. In psychiatry, subtle distinctions in the documentation of risk-related information, particularly regarding suicidality, functional impairment, and protective factors, carry significant clinical consequences. Our findings suggest that overly verbose or templated documentation, as reflected in higher redundancy and altered topic structure, may affect the relative prominence of different types of information within narrative notes, which could be relevant when considering downstream interpretation in safety-critical contexts.

From an educational perspective, the increasing availability of LLM-assisted documentation raises hypotheses regarding how trainees and early-career clinicians might engage with clinical documentation. Specifically, there is a possibility that increased exposure to template-like or highly standardized notes could influence documentation practices, an effect that warrants direct empirical examination rather than inference from the present findings. When clinical notes emphasize surface completeness over effective information integration, there is a possibility that documentation practices become learned primarily through stylistic exposure. One open question for future research is whether extensive exposure to LLM-generated documentation might shape how clinical reasoning and context-sensitive practices are conceptualized during training, an issue that cannot be addressed by the present descriptive analysis. These findings underscore the need for careful governance of LLM use in psychiatric documentation, as well as explicit educational guidance to ensure that narrative notes continue to reflect clinically relevant documentation rather than automated textual patterns.

### Limitations

This study has several limitations. First, it is based on only 2 standardized psychiatric scenarios (depression and schizophrenia), which restricts the generalizability of the findings to other psychiatric conditions, levels of clinical complexity, and real-world settings. Although these scenarios were intentionally designed to enable controlled comparisons, they do not capture the full diversity of clinical presentations, longitudinal disease trajectories, or comorbid conditions encountered in routine practice. As a result, the use of relatively prototypical and simplified scenarios may have contributed to stylistic convergence across clinicians and models, while potentially attenuating differences that might emerge in more complex or ambiguous clinical cases.

Second, the standardized case scenarios were initially drafted with the assistance of an LLM and subsequently reviewed and refined by the authors. Although careful efforts were made to ensure clinical plausibility and internal consistency and to remove unnatural or model-specific phrasing, it is possible that subtle linguistic or structural characteristics originating from the initial LLM drafting process remained. Such residual influences may have shaped the language or structure of the scenarios in ways that could advantage certain model outputs or influence how participating physicians documented their notes, even though physicians were explicitly instructed to freely modify, supplement, or correct the scenarios as needed. Accordingly, this potential source of bias cannot be fully excluded and should be considered when interpreting the results. In addition, because physicians were informed that the scenarios were generated with LLM assistance, this awareness may have influenced their documentation behavior, for example, by increasing vigilance toward inconsistencies or prompting more deliberate modification of the text. Such expectancy effects cannot be fully excluded.

Third, the sample size of physician-authored notes was limited, and variations in individual documentation practices or institutional conventions may not have been fully captured. Our bootstrap-based sensitivity analyses revealed that the impact of sample size imbalance was metric dependent. For lexical surface-form variation, LLM values fell largely within the range of the downsampled distributions of human-authored notes, suggesting that part of the observed differences in this measure may be attributable to small-sample effects. In contrast, several redundancy-related metrics showed robust differences that persisted even with downsampling, indicating that these patterns cannot be explained by sample size imbalance alone. Accordingly, LLM-related findings should be interpreted descriptively rather than inferentially, with attention to the specific linguistic dimension being examined. Moreover, the substantial imbalance between physician-authored notes (n=118) and LLM-generated notes (n=8 in total) may affect the stability of clustering and distribution-based analyses. Accordingly, all group comparisons in this study should be interpreted primarily as a descriptive characterization of stylistic tendencies rather than as confirmatory population-level inferences.

Fourth, this study was conducted using Japanese-language psychiatric documentation, which reflects cultural and institutional conventions specific to the Japanese health care system. In particular, Japanese psychiatric notes often rely on implicit shared clinical understanding and contextual narrative rather than explicitly articulated documentation structure or standardized sectioning. Documentation practices, levels of explicitness, and narrative styles may therefore differ across languages and regions. As such, caution is warranted when generalizing these findings to other health care systems.

Fifth, the LLMs evaluated in this study were general-purpose models and were not fine-tuned on clinical or biomedical corpora. Accordingly, the present findings should be interpreted as characterizing baseline documentation behaviors of off-the-shelf LLMs rather than the maximal capabilities of domain-adapted clinical models. In addition, results were aggregated across multiple LLMs to emphasize general documentation tendencies rather than model-specific behaviors, which may obscure meaningful inter-model variability relevant to downstream clinical or educational applications.

Sixth, although standardized prompts were used, the outputs of LLMs are inherently sensitive to prompt design and configuration. Alternative prompting strategies or model settings may yield different stylistic patterns. Moreover, we did not evaluate iterative or multi-round prompting workflows in which LLM outputs are progressively refined through repeated reprompting or human-in-the-loop revision. The present results should therefore be interpreted as reflecting baseline, single-pass generation behaviors rather than optimized or iteratively edited outputs.

Future studies should therefore include a broader range of clinical cases, documentation contexts, and prompt designs to further validate the robustness and generalizability of these findings.

### Conclusions

This study conducted a multifaceted analysis of the differences in documentation styles among psychiatrists, internists, and LLMs for identical clinical cases, elucidating both the discipline-specific characteristics and the limitations of LLM-based reproduction.

Psychiatric records were characterized by rich emotional descriptions, incorporation of patient background, and a broad range of lexical expressions, demonstrating greater structural and lexical diversity compared with internal medicine records. While LLM outputs were capable of partially mimicking certain stereotypical documentation patterns, they remained prone to redundancy and rigidity of expression.

For the future application of LLMs in medical documentation support, it will be essential to go beyond superficial style imitation and to implement output control that accounts for the intentions of discipline-specific documentation and the clinical context while also ensuring pragmatic diversity. In domains such as psychiatric records—where a wide variety of expressions and nonstandardized information are prevalent—the auxiliary use of LLMs will require cautious design and careful human oversight.

Importantly, this study does not claim that current LLMs are ready for autonomous clinical documentation or unsupervised clinical use. Rather, the present findings are intended to delineate stylistic and structural characteristics under controlled conditions, and should not be interpreted as evidence of clinical safety, diagnostic validity, or deployment readiness.

## Supplementary material

10.2196/85671 Multimedia Appendix 1 English translations of representative case scenarios, prompts, and example clinical notes (reference purposes only); supplementary figures depicting hierarchical clustering of clinical notes; and supplementary table showing top term frequency-inverse document frequency (TF-IDF)–weighted terms by group for the schizophrenia case.

## Abbreviations

BERT: bidirectional encoder representations from transformers
BLEU: Bilingual Evaluation Understudy
IRB: institutional review board
LDA: latent Dirichlet allocation
LLM: large language model
NAIST: Nara Institute of Science and Technology
NLP: natural language processing
ROUGE-L: Recall-Oriented Understudy for Gisting Evaluation–Longest Common Subsequence
TER: Translation Edit Rate
TF-IDF: term frequency-inverse document frequency
TTR: type-token ratio
