# Supplementary material for: Comparative Analysis of Japanese Clinical Note Styles Between Physicians and Large Language Models Using Identical Psychiatric Cases: Quantitative Text Analysis
Source: JMIR Form Res. 2026 Mar 27;10:e85671. doi: 10.2196/85671 (PMC13027678; doi:10.2196/85671)
Supplement: Multimedia Appendix 1 [file formative-v10-e85671-s001.docx]

**Appendix A. Scenarios**

- **A1. Major Depressive Disorder Case (Scenario)
  Case Summary**
  A 45-year-old male office worker, living with his wife and child. About six months after being promoted to section manager, he developed initial insomnia and began making careless mistakes at work. Later, he developed dizziness, fatigue, and loss of appetite. He presented to the clinic on the recommendation of his company physician. He is in a depressive state with mild suicidal ideation.

**Outpatient Initial Psychiatric Interview (Dialogue Format)**

*Characters*

- - **Psychiatrist (Dr):** Provides supportive psychotherapy.
  - **Patient (Pt):** 45-year-old male, office worker.
  - **Wife (W):** Accompanies patient for support.

**Chief Complaint and Present Illness**

- - Dr: “What troubles you the most today?”
  - Pt: “I can’t sleep well, and it’s affecting my work. I make mistakes.”
  - Dr: “Since when?”
  - Pt: “About six months after being promoted.”
  - Pt describes progression: initial insomnia → dizziness, fatigue, loss of appetite.
  - Pt: “Every day I feel drained after work, can’t do anything.”
  - W: “At home too, he’s listless and uninterested.”

**Mental Status Examination**

- - Pt: “My mood is low, I find no pleasure in anything.”
  - Pt: “I feel anxious thinking about work.”
  - Sleep: initial insomnia, occasional nocturnal awakening.
  - Appetite: markedly reduced, with weight loss.
  - Motivation: significantly decreased.
  - Dr: “Do you ever feel like dying?”
  - Pt: “Sometimes I wish everything would end, but I have no specific plan.”
  - No history of manic/hypomanic episodes.

**Physical Symptoms and Patient’s Model**

- - Pt: “Constant fatigue. I think it’s stress from responsibility.”

**History**

- - Normal development, strict father.
  - No significant illness or injuries.
  - No current medications.

**Family, Education, Career**

- - No family psychiatric history.
  - University graduate, employed at same company >20 years.

**Premorbid Personality and Treatment Expectation**

- - Pt: “I’m responsible but perfectionistic, which is burdensome.”
  - Pt: “I just want to recover and work normally again.”

**Diagnosis and Treatment Plan**

- - Dr: “You are experiencing a depressive episode, with stress as a major factor. Since mild suicidal ideation is present, early treatment is necessary. We will start an SSRI, e.g., sertraline 25–50 mg/day, and combine with psychotherapy and stress management.”
  - Pt: “If it helps, I’ll take it.”

**Closing**

- - Pt and W express desire for recovery and support.
  - Dr emphasizes importance of family involvement.
- **A2. Schizophrenia Case (Scenario)
  Case Summary**
  A 21-year-old male, accompanied by his parents, was brought to the hospital due to strange behavior. Since taking his graduate school entrance examination a week ago, he has been unable to sleep. On the morning of presentation, he was found walking in circles around a bench near the station for about three hours while talking to himself, and was taken into protective custody by the police. On arrival, he showed poor gestures and facial expressions, avoided eye contact, sometimes smiled inappropriately, and at other times appeared fearful. He occasionally nodded as if listening to something and suddenly muttered phrases such as “I see” or “That explains it.” Developmental history, adaptation, blood chemistry, brain MRI, and EEG were all unremarkable.

**Outpatient Initial Psychiatric Interview (Dialogue Format)**

*Characters*

- - **Psychiatrist (Dr):** Provides supportive psychotherapy.
  - **Patient (Pt):** 21-year-old male, university student.
  - **Father (F):** Accompanies patient for support.
  - **Mother (M):** Accompanies patient for support.

**Chief Complaint and Present Illness**

- - Dr: “Thank you for coming today. First, may I ask you, the parents, about the patient’s condition?”
  - F: “He has been unable to sleep since about a week ago, after his graduate school entrance exam. Today, he was found walking around a bench near the station, talking to himself for hours, and was taken in by the police.”
  - M: “He used to be a quiet child, but suddenly started acting strangely. We were shocked.”
  - Dr: “When did these behaviors begin specifically?”
  - F: “Right after the exam. Before that, nothing unusual.”
  - Dr: “How has this affected his daily life or studies?”
  - M: “Before the exam, he was fine. Now, he barely talks and avoids eye contact.”

**Mental Status Examination**

- - Dr: “Let me ask the patient directly. How have you been feeling recently?”
  - (Patient remains silent, avoids eye contact, occasionally nods as if listening.)
  - Pt: (suddenly) “I see… that explains it.”
  - Dr: “Is something troubling you?”
  - (No answer, continues staring.)
  - Dr (to parents): “Has he ever talked to himself before?”
  - F: “No, never before this week.”
  - M: “This started suddenly.”
  - Dr: “How about appetite?”
  - M: “Almost none. He seems to have lost some weight.”
  - Dr: “Any signs of anxiety or depression?”
  - F: “Rather, he shows a blank face, suddenly smiling or looking scared.”
  - Dr: “Do you feel like you’re being watched or monitored?”
  - Pt: (suddenly) “Being watched? Well, that may be true.”
  - Dr: “By whom, specifically?”
  - (No response.)

**Physical Symptoms and Patient’s Model**

- - Dr: “Do you have any physical pain or discomfort?”
  - (No response.)
  - Dr: “Anything you noticed yourself?”
  - Pt: (shortly) “Don’t know.”

**History**

- - M: “No developmental or adaptation problems.”
  - F: “No significant illnesses or injuries.”
  - M: “No current or past medications.”

**Academic/Work and Premorbid Personality**

- - F: “He is a university student, preparing for graduate school.”
  - M: “He is diligent, quiet, and somewhat introverted.”

**Diagnosis and Treatment Plan**

- - Dr: “From what I see, schizophrenia is suspected—insomnia, hallucination-like symptoms, and strange behavior. We will start medication and psychosocial support.”
  - F: “What medication?”
  - Dr: “Atypical antipsychotic, such as risperidone, starting at 1 mg/day.”
  - M: “Are side effects a concern?”
  - Dr: “We will monitor carefully.”

**Closing**

- - Parents express concern.
  - Dr emphasizes importance of family support.

**Appendix B. Prompts Provided to the LLM**

- You are a psychiatrist/internist.
  Based on the provided scenario, please create a psychiatric outpatient initial consultation note (summary).
  The format may follow the conventions of the institution where you practice.
  *If there are any missing elements in the scenario (e.g., differential diagnoses, questions that should be asked for treatment planning) or unnatural parts, please supplement or revise them as appropriate based on your clinical experience and reflect them in the note.*

**Appendix C. LLM-Generated Clinical Notes**

- **Major Depressive Disorder Case (Generated Note)**Model used: *ChatGPT-4o (OpenAI)*
  Role: *Psychiatrist* **Basic Information**
  - Name: [Anonymized]
  - Age: 45 years
  - Sex: Male
  - Occupation: Company employee (section manager)
  - Accompanied by: Wife

**Chief Complaint**
“Difficulty falling asleep, leading to increased mistakes at work.”

**History of Present Illness**
Approximately six months ago, after being promoted to section manager, the patient began experiencing difficulty initiating sleep. Gradually, careless mistakes at work became more noticeable. Subsequently, dizziness, fatigue, and loss of appetite developed, leading him to seek consultation upon recommendation from the occupational physician.
He presents with depressed mood, anhedonia, decreased motivation, and mild suicidal ideation. Sleep disturbance is characterized mainly by difficulty falling asleep, occasionally accompanied by middle insomnia. Appetite is markedly reduced, with associated weight loss. Daily functioning is impaired, and his interest in family has diminished.
He has strong self-blame, attributing it to his sense of responsibility and perfectionistic tendencies.

**Past Medical History**
No notable medical history.

**Family History**
No family members with psychiatric disorders.

**Developmental History**
No developmental problems.

**Education and Work History**
Graduated from university and has worked at the same company for over 20 years. Stress increased following promotion to section manager.

**Premorbid Personality**

- - Strong sense of responsibility, perfectionistic tendencies.
  - Low tolerance for failure (influenced by strict father).

**Life History**

- - Lives with wife and child (three-person household).

**Mental Status Examination**

- - Appearance: Appropriate for age, good hygiene.
  - Consciousness: Clear.
  - Attention and concentration: Slightly impaired.
  - Thought: Self-blame associated with depressed mood; no thought blocking or flight of ideas.
  - Affect: Depressed.
  - Motivation: Decreased.
  - Delusions/hallucinations: None observed.
  - Suicidal ideation: Mild, without specific plans or behaviors.
  - Sleep: Difficulty initiating sleep, occasional middle insomnia.
  - Appetite: Markedly decreased.
  - Fatigue/somatic complaints: General fatigue, dizziness.
  - Anxiety: Related to work.
  - History of hypomania: Negative.
  - Impulsivity/irritability: Negative.
  - Physical Examination
  - General condition: No abnormal findings. (Consider internal medicine screening as needed.)

**Provisional Diagnosis**
Major Depressive Disorder, Single Episode, Moderate severity

**Differential Diagnosi**s

- - Adjustment disorder (excluded due to fulfillment of major depressive episode criteria).
  - Bipolar disorder (negative history of hypomanic episodes).
  - Secondary depression due to medical illness (internal medicine screening required).
  - Primary sleep disorder (alone insufficient to explain current symptoms).

**Treatment Plan**

- - Pharmacotherapy: Initiate SSRI (e.g., sertraline starting at 25 mg/day, with gradual titration).
  - Psychotherapy: Supportive psychotherapy with stress management guidance.
  - Occupational considerations: Explore collaboration with employer regarding leave or workload reduction.
  - Family support: Provide wife with advice on understanding and supporting the patient.
  - Suicide risk management: Continue to monitor the progression of suicidal ideation.

**Patient and Family Education**
Explained that current symptoms are due to depression and can be expected to improve with treatment.
Discussed potential side effects of medication (e.g., nausea, fatigue) and the rationale for starting at a low dose.
Emphasized the importance of family support.
Instructed to seek immediate consultation or emergency care if suicidal ideation worsens.

- **Major Depressive Disorder Case (Generated Note)**Model used: *ChatGPT-4o (OpenAI)*
  Role: *internist***Patient Information**
  - Age/Sex: 45-year-old male
  - Occupation: Office worker (section manager)
  - Accompanied by: Wife

**Chief Complaint**
Difficulty falling asleep, increased mistakes at work, fatigue, and loss of appetite.

**History of Present Illness**
Approximately six months ago, following his promotion to section manager and the associated increase in job responsibilities, the patient became aware of difficulty initiating sleep. Subsequently, careless mistakes at work became more noticeable. Gradually, he developed dizziness, generalized fatigue, and decreased appetite. Recently, weight loss has also been observed.

He visited our department upon the recommendation of an occupational physician.

During the daytime, he experiences decreased concentration and marked fatigue, and after returning home, he is often unable to do anything and lies down.
At home, his interest in household matters and his children has diminished, and his wife has pointed out these changes.

He reports a depressed mood, loss of interest, and decreased motivation. He also endorses mild suicidal ideation (described as “feelings of wanting everything to end”), but denies any specific suicide plans or history of attempts.

Mild anxiety symptoms related to work are noted, but he denies agitation or panic attacks.
There are no episodes suggestive of bipolar disorder, such as elevated mood, irritability, or impulsive spending behaviors.

**Past Medical History**
No significant past medical history

**Medication History**
No current medications

**Allergy History**
No known allergies

**Family History**
No relatives with psychiatric disorders

**Developmental History**
Within normal range

**Social History**

- Employed at the same company for over 20 years since graduating from university
- Lives with his spouse and child (household of three)

**Premorbid Personality**

- - Strong sense of responsibility, perfectionistic tendencies
  - Self-critical in response to failure

**Physical Examination (on the day of visit)**

- - General condition: Clear consciousness, vital signs stable
  - No abnormal findings on physical examination suggestive of organic disease

*Additional tests such as blood tests, thyroid function tests, and brain imaging may be considered as needed.*

**Mental Status Examination (Summary)**

- - Depressed mood, decreased motivation, loss of interest, mild suicidal ideation
  - Mild psychomotor retardation; no delusions or hallucinations in thought content
  - Decreased attention and concentration
  - Sleep disturbance (mainly difficulty initiating sleep with a tendency toward middle-of-the-night awakenings)
  - Decreased appetite and weight loss

**Differential Diagnosis**

- - Major Depressive Disorder (MDD)
  - Adjustment Disorder (as stress-related symptoms; however, not the primary diagnosis given the severity of symptoms)
  - Bipolar Disorder (currently denies episodes of hypomania or mania)
  - Organic disease (secondary depressive symptoms due to thyroid dysfunction, anemia, malignancy, etc., should be ruled out)

**Initial (Provisional) Diagnosis**

Major Depressive Disorder, Moderate

**Initial Treatment Plan**

**Pharmacotherapy:**

- - SSRI (e.g., start sertraline at 25 mg/day, with gradual titration)
  - Monitoring for side effects (e.g., gastrointestinal symptoms, activation syndrome)

**Psychotherapy:**

- - Supportive psychotherapy (consider collaboration with a clinical psychologist)
  - Stress coping skills training

**Sick Leave / Workload Reduction:**

- - Coordination with the occupational physician and workplace (consider issuing a medical certificate if necessary)

**Suicide Risk Management:**
Continued risk assessment, with instructions for emergency intervention if symptoms worsen

**Explanation to the Patient and Family**

- - Explained the nature of the illness and its treatability.
  - Provided guidance on the purpose of antidepressant treatment, potential side effects, and the importance of medication adherence.
  - Emphasized the importance of monitoring and support by the family.
- **Schizophrenia Case (Generated Note)**Model used: *ChatGPT-4o (OpenAI)*
  Role: *Psychiatrist*

**Basic Information**
• Name: [Anonymized]
• Age: 21 years
• Sex: Male
• Occupation: University student (recently took a graduate school entrance examination)

**Chief Complaint**
Bizarre behavior (wandering while talking to himself), insomnia, and difficulty engaging in conversation.

**History of Present Illness**
Approximately one week ago, following a graduate school entrance examination, the patient began experiencing marked insomnia.
Subsequently, abnormal behaviors became prominent, including talking to himself, aimless walking, reduced facial expressiveness, and avoidance of eye contact.

Today, he was found wandering near a bench in front of a train station for approximately three hours while talking to himself, after which he was taken into protective custody by the police and brought to the hospital accompanied by his parents.

Meaningful conversation could not be established, and his responsiveness to the external environment was poor. At times, he stared fixedly at a single point, suddenly smiled inappropriately, or displayed frightened facial expressions, indicating abnormal emotional expression.

Although his responses to questions were generally limited, he occasionally uttered incomprehensible remarks such as “I see” or “That’s why,” in a soliloquizing manner.

He also exhibited behaviors suggestive of auditory hallucinations, such as appearing to listen attentively and nodding.
Spontaneous provision of information was minimal, making it difficult to obtain detailed insight into his internal experiences.

The onset was acute. Premorbidly, he had been functioning well academically, but a sudden emergence of psychiatric symptoms was observed.

**Past Medical History**
• No notable medical or psychiatric history

**Life History and Developmental History**
• Normal developmental history
• Good adaptation to school life since childhood

**Family History**
• No family members with psychiatric disorders

**Personality Traits (Premorbid Personality)**
• Introverted, quiet, and serious personality
• Slightly low sociability

**Medication History and Allergy History**
• No notable history

**Physical and Laboratory Findings**
• No abnormalities on general physical examination
• No abnormal findings on blood biochemistry tests, brain MRI, or EEG

**Mental Status Examination**
• Consciousness: Clear; orientation appears intact (although difficult to confirm in some situations)
• Appearance: Reduced facial expression, alternating between flat affect and abnormal emotional expressions (inappropriate smiling, fearfulness)
• Speech: Presence of soliloquy; minimal response to questions
• Thought: Suggestive of thought blocking–like phenomena and disorganized thinking
• Perception: Strongly suspicious for auditory hallucinations (passivity phenomena)
• Affect: Emotional blunting and unstable emotional expression
• Behavior: Aimless wandering, stereotyped-like behaviors
• Suicidal or homicidal ideation: No clear intent at present (however, caution is warranted due to unclear internal experiences)

**Differential Diagnosis**

Schizophrenia (acute phase)

Acute and transient psychotic disorder (brief psychotic disorder)

Mood disorder with psychotic features (e.g., manic psychosis)

Substance-induced psychotic disorder (currently denies substance use)

Organic mental disorder (no evident physical or neurological disease)

**Provisional Diagnosis**
• Schizophrenia, acute phase

**Initial Treatment Plan**

• **Pharmacotherapy:**
o Atypical antipsychotic (e.g., start risperidone at 1 mg/day, with gradual titration)
o Monitoring for side effects (extrapyramidal symptoms, weight gain, metabolic abnormalities, etc.)

• **Indication for Hospitalization:**
o Voluntary consent for treatment is difficult; consideration of involuntary hospitalization under designated mental health legislation or medical protective admission

• **Psychosocial Interventions:**
o Psychoeducation for the family after symptom stabilization
o Support for developing the patient’s insight into the illness

**Explanation to the Family**
• Provided an explanation of the characteristics and course of schizophrenia
• Explained that the primary goal of initial treatment is to control hallucinations, delusions, and abnormal behaviors
• Emphasized that long-term goals include medication adherence, relapse prevention, and recovery of social functioning
• Explained that initial treatment requires several weeks and that patience is necessary
• Provided advance explanation regarding legal procedures in case hospitalization becomes necessary (e.g., medical protective admission)

- **Schizophrenia Case (Generated Note)**Model used: *ChatGPT-4o (OpenAI)*
  Role: *Internist*

**Patient Information**
• Name: [Anonymized]
• Age: 21 years
• Sex: Male
• Occupation: University student (planning to enter graduate school)

**Chief Complaint**
Bizarre behavior, insomnia, and talking to himself.

**History of Present Illness**
Approximately one week ago, after taking a graduate school entrance examination, the patient began complaining of insomnia.
For several days, he was unable to sleep at night and appeared to have poor concentration during the daytime.

This morning, he was found wandering around a bench near a train station for approximately three hours while talking to himself. He was taken into protective custody by the police and brought to the hospital accompanied by his parents.

At the time of presentation, the patient showed poor responsiveness to the external environment, with limited gestures and facial expressions.
At times, he suddenly smiled inappropriately or displayed frightened facial expressions, indicating emotional instability.

His responses to questions were minimal; however, he occasionally uttered short, irrelevant soliloquies such as “I see” or “That makes sense,” which were unrelated to the surrounding context.

In addition, his gaze was unstable, and he sometimes stared fixedly at a single point or showed excessive vigilance toward his surroundings.

Premorbidly, he had no notable developmental delays or academic difficulties and was described as serious and introverted.

**Past Medical History**
• No notable past medical conditions

**Life History and Developmental History**
• Normal developmental history
• Good adaptation during early childhood and school age

**Family History**
• No family members with psychiatric disorders

**Medication History**
• None

**Allergy History**
• None

**Current Status (Physical and Behavioral Findings)**
• Consciousness: Clear
• Gaze: Tendency toward fixed staring; difficulty making eye contact
• Facial expression: Generally limited, with occasional sudden laughter or fearful expressions
• Speech: Presence of soliloquy; minimal responses to questions
• Behavior: Inappropriate behaviors for the situation (tendency toward aimless wandering)
• Physical findings: No abnormalities (stable blood pressure and pulse; afebrile)

**Laboratory and Imaging Results**
• Blood biochemistry tests: No abnormal findings
• Brain MRI: No abnormal findings
• Electroencephalogram (EEG): No abnormal findings

**Differential Diagnosis**

Schizophrenia, acute phase (first-episode psychosis)

Acute and transient psychotic disorder

Mood disorder with psychotic features (e.g., manic episode with psychosis)

Substance-induced psychotic disorder (no information suggesting substance use at present)

Organic mental disorder (considered unlikely based on test results)

*At present, physical causes such as organic disease, metabolic disorders, or infections are considered unlikely.*

**Provisional Diagnosis**
• Suspected psychotic disorder (suspected schizophrenia)

**Initial Management and Treatment Plan**
• Plan for early referral to a psychiatric specialist
• Close monitoring for worsening psychiatric symptoms and risk of self-harm or harm to others
• Explained that a psychiatric emergency visit accompanied by a guardian is also an option
• If necessary, hospitalization under the Mental Health and Welfare Act (e.g., medical protective admission) will be considered

**Explanation to the Family**
• Explained that the symptoms are highly likely to be due to a psychiatric disorder and require specialized treatment
• Explained the possibility that acute-phase inpatient treatment may become necessary depending on the clinical course
• Instructed the family to seek immediate medical attention or emergency services if the patient’s condition acutely worsens (e.g., emergence of violent behavior, self-harm, or homicidal ideation)

- **Major Depressive Disorder Case (Generated Note)**Model used: *Claude Sonnet4 (Anthropic)*
  Role: *Psychiatrist***Patient ID:** 111B47
  **Date of Visit:** XX, 2024
  **Department:** Psychiatry
  **Physician:** XXX

**Patient Information**

• Name: XXX
• Age/Sex: 45-year-old male
• Occupation: Office worker (section manager)
• Family structure: Lives with wife and child (household of three)
• Referral source: Occupational physician
• Accompanied by: Wife

**Chief Complaint**

“I have difficulty falling asleep, and I am making more mistakes at work.”

**History of Present Illness**

Approximately six months after being promoted to section manager, the patient developed difficulty initiating sleep, followed by a gradual worsening of symptoms. He currently presents with depressed mood, loss of interest and motivation, dizziness, generalized fatigue, loss of appetite, and weight loss. The increase in careless mistakes at work has further exacerbated his stress. He reports mild passive suicidal ideation but denies any specific suicide plans. He visited our clinic upon the recommendation of his occupational physician.

**Course since onset (approximately 6 months):**
• Six months after promotion: Onset of difficulty initiating sleep
• Gradual worsening: Development of dizziness, fatigue, and appetite loss
• Current: Persistent daily symptoms, decline in social functioning, apathy also observed at home

**Mental Status Examination**

**Appearance and Attitude**

• Cooperative, approachable demeanor
• Facial expression mildly depressed but with appropriate emotional expression
• Attended with his wife; family support appears strong

**Mood and Affect**

• Depressed mood: Persistent low mood
• Loss of interest: Complete loss of interest in previously enjoyed hobbies
• Anhedonia: “Nothing feels enjoyable anymore”
• Anxiety: Present, mainly related to work
• Agitation: Denied

**Sleep**

• Difficulty initiating sleep: Primary symptom, persistent every night
• Middle-of-the-night awakening: Occasional, with difficulty returning to sleep
• Early morning awakening: Requires further evaluation
• Daytime sleepiness: Requires further assessment

**Appetite and Weight**

• Loss of appetite: Marked (“I hardly feel like eating anything”)
• Weight loss: Mild (subjectively reported; objective measurement required)

**Motivation and Activity**

• Decreased motivation: Marked (“Even daily activities feel burdensome”)
• Psychomotor retardation: Mild to moderate
• Easy fatigability: “I suddenly feel exhausted and can’t do anything”

**Cognitive Function**

• Decreased concentration: Increase in careless mistakes at work
• Indecisiveness: Requires further evaluation
• Memory impairment: No clear complaints

**Suicidal Ideation and Suicide Risk**

• Passive suicidal ideation: Mild (“I want everything to end”)
• Active suicidal ideation: No specific plans
• Access to means: Requires further assessment
• Protective factors: Strong family support

**Screening for Manic Symptoms (Including Past History)**

• Elevated mood, increased activity, decreased need for sleep, pressured speech, grandiosity, irritability, disinhibited behavior: All denied
• No episodes suggestive of mania reported by the wife
• Hypomanic episodes: Denied

**Psychotic Symptoms**

• Hallucinations/delusions: Denied
• Thought disorder: Denied

**Physical Symptoms and Medical Evaluation**

**Physical Symptoms**

• Dizziness
• Generalized fatigue (“I never feel rested”)
• Loss of appetite
• Mild weight loss

**Necessary Medical Examinations (to be conducted)**

• Blood tests: CBC, biochemistry, thyroid function (TSH, fT3, fT4)
• ECG, chest X-ray
• If necessary: Brain MRI, EEG

**Life History and Developmental History**

• Upbringing: Typical household; father was strict and intolerant of failure (possibly contributing to perfectionistic tendencies)
• Developmental history: No notable problems
• Education: University graduate
• Adjustment history: Good

**Past Medical and Medication History**

• Past medical history: None
• Past psychiatric history: None (first episode)
• Current medications: None
• Allergy history: To be confirmed
• Substance use history: Requires detailed assessment (alcohol, drugs, etc.)

**Family History**

• Family history of psychiatric disorders: Denied
• Family history of suicide: To be confirmed

**Occupational and Social History**

• Current employment: Over 20 years at the same company
• Promotion history: Promoted to section manager approximately 6 months ago
• Work adjustment: Good prior to promotion
• Financial status: Presumed stable
• Social support: Good

**Premorbid Personality**

• Strong sense of responsibility
• Marked perfectionism (“Sometimes it becomes a burden”)
• Serious and conscientious (inferred)
• Melancholic temperament: Suggested

**Patient and Family Understanding of the Illness and Treatment Motivation**

• Patient’s understanding: Recognizes that stress from promotion is a contributing factor; appropriate insight
• Motivation for treatment: Good (“I want to be able to do my job properly again”)
• Family understanding: Wife observes symptoms appropriately and is cooperative with treatment
• Treatment expectations: Realistic expectations for functional recovery

**Diagnosis**

**Primary Diagnosis**

• F32.1 Moderate depressive episode (ICD-10)
• 296.22 Major Depressive Disorder, single episode, moderate (DSM-5-TR)

**Diagnostic Rationale (DSM-5-TR Criteria)**

**Criterion A (5 out of 9 symptoms required; patient meets 7–8):**

✓ Depressed mood (nearly every day)

✓ Markedly diminished interest or pleasure (anhedonia)

✓ Loss of appetite and weight loss

✓ Insomnia (difficulty initiating sleep)

✓ Psychomotor retardation

✓ Fatigue or loss of energy

✓ Feelings of worthlessness or excessive guilt (requires further assessment)

✓ Diminished ability to think or concentrate

✓ Suicidal ideation

**Criterion B:** Clinically significant distress and functional impairment
**Criterion C:** Not attributable to substances or a medical condition
**Criterion D:** Not better explained by manic or hypomanic episodes

**Severity Assessment**

• Number of symptoms: 7–8 out of 9
• Functional impairment: Marked (occupational and family functioning)
• Psychotic features: None
• Severity: Moderate

**Specifiers**

• With anxious distress: Mild
• With melancholic features: To be evaluated (e.g., loss of appetite, anhedonia)

**Differential Diagnosis**

**Excluded**

Bipolar disorder – No history of manic or hypomanic episodes

Psychotic disorders – No psychotic symptoms

**To Be Ruled Out**

Depressive disorder due to a medical condition
o Hypothyroidism
o Adrenal dysfunction
o Organic brain disease

Substance-induced depressive disorder
o Detailed history of alcohol and substance use required

Adjustment disorder
o Excluded based on symptom severity and duration

Persistent depressive disorder (dysthymia)
o No chronic course of ≥2 years

**Risk Assessment**

**Suicide Risk (Moderate)**

**Risk Factors:**
• Middle-aged male
• Passive suicidal ideation
• Moderate depressive symptoms
• Perfectionistic personality
• Work-related stress

**Protective Factors:**
• Strong family support
• No specific suicide plan
• Good motivation for treatment
• First episode

**Functional Impairment Risk**

• Occupational functioning: Markedly impaired
• Family functioning: Moderately impaired
• Social functioning: Moderately impaired

**Treatment Plan**

**Pharmacotherapy**

**First-line medication:**
• Sertraline (SSRI)
o Starting dose: 25 mg/day (after dinner) for 1 week
o Maintenance dose: Increase to 50 mg/day
o Maximum dose: Up to 100 mg/day if necessary

**Rationale for medication choice:**
• First-line treatment for moderate depression
• Favorable side-effect profile
• Suitable for middle-aged male patients

**Side effect monitoring:**
• Gastrointestinal symptoms (nausea, diarrhea)
• Sexual dysfunction
• Activation syndrome (rare but requires attention)
• Weight changes

**Efficacy evaluation:**
• Initial response assessment at 4–6 weeks
• Adequate response evaluation at 8–12 weeks

**Psychosocial Interventions**

**Supportive Psychotherapy**
o Establishment of therapeutic alliance
o Normalization of symptoms
o Maintenance of treatment motivation

**Consideration of Cognitive Behavioral Therapy (CBT)**
o Modification of perfectionistic thought patterns
o Enhancement of stress-coping skills
o Support for return to work

**Psychoeducation**
o Improving understanding of the illness
o Enhancing medication adherence
o Education for relapse prevention

**Environmental Adjustment and Social Support**

**Workplace Adjustment**
o Collaboration with the occupational physician
o Consideration of workload modification
o Gradual return-to-work program

**Family Support**
o Education about the illness
o Guidance on appropriate responses
o Reduction of family burden

**Follow-up Plan**

**Short-term Follow-up (1–3 months)**

• 1 week: Check for side effects, adjust dosage
• 2 weeks: Symptom evaluation, adherence check
• 4 weeks: Evaluate efficacy, consider psychotherapy
• 8 weeks: Reassess treatment response, consider timing of return to work

**Mid- to Long-term Follow-up (After 3 months)**

• Monthly: Maintenance treatment during stable phase
• Every 3 months: Comprehensive reassessment
• At 6 months: Consideration of treatment duration

**Treatment Goals**

**Short-term Goals (1–3 months)**

≥50% improvement in depressive symptoms

Normalization of sleep and appetite

Resolution of suicidal ideation

Recovery of basic daily functioning

**Mid-term Goals (3–6 months)**

Achievement of remission

Successful return to work

Recovery of family roles

Improved stress-coping ability

**Long-term Goals (After 6 months)**

Maintenance of full remission

Prevention of relapse

Improvement of quality of life

Consideration of discontinuation of pharmacotherapy

**Prognosis**

**Favorable Prognostic Factors:**
• First episode
• Clear precipitating factor (promotion-related stress)
• Good premorbid adjustment
• Strong family support
• High treatment motivation

**Factors Requiring Attention:**
• Perfectionistic personality
• Onset in midlife
• Ongoing workplace stress

**Expected Course:**
With appropriate treatment, symptom improvement is expected within 6–8 weeks, and remission may be achieved within 3–6 months. Maintenance therapy for 12–18 months is recommended to prevent relapse.

**Special Notes and Considerations**

**Suicide Risk Management**
o Regular assessment of suicidal ideation
o Emergency response guidance for family
o Establishment of a contact system for symptom worsening

**Support for Return to Work**
o Close collaboration with the occupational physician
o Gradual return-to-work program
o Need for workplace accommodations

**Management of Perfectionism**
o Correction of cognitive distortions
o Support for realistic goal setting
o Acquisition of stress management techniques

**Consideration for Family**
o Promotion of accurate understanding of the illness
o Reduction of family burden
o Strengthening collaboration in treatment

**Next Appointment**

One week later – Assessment of side effects and symptom evaluation after initiation of pharmacotherapy

**Physician’s Signature:** XXX
**Date of Entry:** XX, 2024

- **Major Depressive Disorder Case (Generated Note)**Model used: *Claude Sonnet4 (Anthropic)*
  Role: *Internist***Patient ID:** 111B47
  **Date of Visit:** XX, 2024
  **Department:** Psychiatry
  **Physician:** XXX

**Patient Information**

• Name: XXX
• Age/Sex: 45-year-old male
• Occupation: Office worker (section manager)
• Family structure: Lives with wife and child (household of three)
• Referral source: Occupational physician

**Chief Complaint**

“I have difficulty falling asleep, and I am making more mistakes at work.”

**History of Present Illness**

Approximately six months after being promoted to section manager, insomnia (difficulty initiating sleep) developed. Subsequently, careless mistakes at work increased, followed by the sequential onset of dizziness, fatigue, and loss of appetite. Symptoms persist daily, and after returning home he is so markedly fatigued that he is unable to do anything. Decreased work efficiency and an increased number of mistakes have become additional stressors. He presented for evaluation upon the recommendation of his occupational physician.

**Timeline:**
• Six months after promotion: Onset of difficulty initiating sleep
• Thereafter: Increase in careless mistakes
• Current: Persistent symptoms with additional dizziness, fatigue, and loss of appetite

**Mental Status Examination**

**Mood and Affect**

• Depressed mood: Persistent low mood
• Loss of interest: Loss of interest in previously enjoyed hobbies
• Anxiety: Work-related anxiety present
• Agitation: Not clearly present

**Sleep**

• Difficulty initiating sleep: Every night
• Middle-of-the-night awakening: Occasional, with difficulty returning to sleep

**Appetite and Weight**

• Loss of appetite: Marked
• Weight loss: Mild (subjectively reported by the patient)

**Motivation and Activity**

• Decreased motivation: Marked; daily activities generally feel burdensome
• Psychomotor retardation: Mild

**Cognitive Function**

• Decreased concentration: Increase in careless mistakes at work
• Memory impairment: Not clearly present

**Suicidal Ideation**

• Mild passive suicidal ideation (“I want everything to end”)
• Specific suicide plan: None
• Suicide risk assessment: Moderate

**Screening for Manic Symptoms**

• Elevated mood, increased activity, decreased need for sleep, pressured speech, irritability, disinhibited behavior, etc.: All denied
• No episodes suggestive of mania reported by the wife

**Physical Symptoms**

• Dizziness
• Generalized fatigue
• Loss of appetite
• Other physical symptoms: No notable findings

**Life History and Developmental History**

• Upbringing: Typical household; father was strict and had a harsh attitude toward failure
• Development: No notable problems
• Education: University graduate

**Past Medical and Medication History**

• Past medical history: No notable history
• Current medications: None
• Allergy history: Unknown (to be confirmed)

**Family History**

• Family history of psychiatric disorders: None

**Occupational and Social History**

• Current workplace: Employed for over 20 years
• Promotion history: Section manager (approximately 6 months ago)
• Before promotion: No particular problems
• Alcohol use history: To be confirmed
• Smoking history: To be confirmed

**Premorbid Personality**

• Strong sense of responsibility
• Perfectionistic tendencies
• Serious and meticulous (inferred)

**Patient and Family Understanding and Expectations**

• Patient’s understanding of illness: Recognizes that pressure and stress related to promotion are contributing factors
• Expectations for treatment: Recovery of physical condition and ability to work without impairment
• Family situation: Wife is concerned about the patient’s apathy and decreased interest in household duties and childcare; cooperative with treatment

**Diagnosis**

**Primary diagnosis:** F32.1 Moderate depressive episode (ICD-10)
**DSM-5-TR diagnosis:** 296.22 Major Depressive Disorder, single episode, moderate

**Conditions to be excluded in the differential diagnosis:**
• Bipolar disorder (no history of manic episodes)
• Adjustment disorder (excluded based on symptom severity and duration)
• Mood disorder due to a medical condition (e.g., thyroid dysfunction)
• Substance-induced mood disorder

**Severity and Risk Assessment**

• Depressive symptoms: Moderate
• Suicide risk: Moderate (passive suicidal ideation present; no plan; family support present)
• Social functioning: Markedly impaired (reduced work efficiency and decreased functioning at home)

**Treatment Plan**

**Pharmacotherapy**

• First-line: SSRI (sertraline)
o Starting dose: 25 mg/day (after dinner)
o After 1 week: Increase to 50 mg/day if well tolerated
o Target dose: 50–100 mg/day
• Side effect monitoring: Gastrointestinal symptoms, sexual dysfunction, activation syndrome, etc.
• Efficacy evaluation: Assess at 4–6 weeks

**Psychosocial Treatment**

• Supportive psychotherapy
• Consideration of cognitive behavioral therapy
• Stress management guidance
• Consideration of workplace accommodations (collaboration with the occupational physician)

**Follow-up Plan**

• First follow-up: In 1 week (check side effects, adjust dose)
• Thereafter: Follow-up at 2-week intervals
• After improvement: Follow-up at 4-week intervals

**Special Notes and Points of Caution**

Suicide risk management: Continue monitoring suicidal ideation; provide information to the family and maintain coordination

Caution at initiation of pharmacotherapy: Risk of activation syndrome in younger patients (low risk at age 45, but caution warranted)

Support for return to work: Collaboration with the occupational physician; consider a gradual return-to-work program

Family support: Provide education to the wife regarding the illness; consider strategies to reduce the family burden

**Next Appointment**

In 1 week, for side-effect assessment and dose adjustment

**Attending physician signature:** XXX
**Date of entry:** XX, 2024

- **Schizophrenia Case (Generated Note)**Model used: *Claude Sonnet4 (Anthropic)*
  Role: *Psychiatrist*

**Patient ID:** 112D35
**Date of Visit:** XX, 2024
**Department:** Psychiatry
**Physician:** Dr. ○○○○

**Patient Information**

• Name: ○○ ○○
• Age: 21 years
• Sex: Male
• Occupation: University student (planning to apply to graduate school)
• Mode of presentation: Brought in by parents after being taken into protective custody by the police

**Chief Complaint**

“Because he is exhibiting bizarre behavior” (as reported by his parents)

**History of Present Illness**

**Onset:** One week ago (immediately after taking the graduate school entrance examination)

**Symptom course:**
• Persistent insomnia since the examination
• This morning, he was found wandering near a bench in front of a train station for approximately three hours while talking to himself and was taken into protective custody by the police
• Avoids eye contact; paucity of gestures and facial expressions
• Fixed staring; abrupt changes in facial expression (inappropriate smiling → fearful expression)
• Auditory hallucination–like symptoms (appearing to listen to something, nodding)
• Soliloquy: dialog-like utterances such as “I see” and “That makes sense”
• Lack of response to questions
• Decreased appetite and weight loss
• Complaints of feeling watched

**Precipitating factor:** Stress related to the graduate school entrance examination

**Past Medical and Psychosocial History**

• Past medical history: No notable history
• Developmental history: Normal development; no problems with adaptation
• Educational background: Currently enrolled in university; good academic performance
• Premorbid personality: Introverted, serious, diligent
• Family history: Family history of psychiatric disorders to be confirmed
• Substance use history: Alcohol and drug use history to be confirmed

**Medication and Allergy History**

• Current medications: None
• Allergy history: No notable history

**Mental Status Examination**

**Appearance and Behavior**

• Appearance appropriate for age
• Paucity of gestures and facial expressions
• Avoids eye contact; tendency toward fixed staring
• Able to maintain a seated position

**Consciousness and Orientation**

• Consciousness: Clear
• Orientation: Requires detailed assessment

**Thought**

• Thought process: Possible disorganized thinking
• Thought content: Suspicion of delusion of being watched
• Soliloquy: Presence of dialog-like self-talk

**Perception**

• Auditory hallucinations: Suspected based on listening behavior and dialog-like soliloquy, possibly command or conversational hallucinations
• Visual hallucinations: No clear symptoms

**Affect and Mood**

• Emotional blunting
• Inappropriate affect (abrupt changes in facial expression)

**Motivation and Spontaneity**

• Decreased motivation
• Decreased spontaneity

**Insight**

• Lacks insight into illness

**Physical Examination**

• Vital signs: Stable (detailed documentation required)
• Neurological findings: No obvious abnormalities
• General physical findings: No remarkable abnormalities except for weight loss

**Laboratory and Imaging Findings**

• Blood chemistry: No abnormalities
• Brain MRI: No abnormalities
• EEG: No abnormalities
• Urine drug screening: Planned
• Thyroid function tests: Planned

**Differential Diagnosis**

**Primary Consideration**

**Schizophrenia (F20)**
• Auditory hallucinations, delusions, disorganized thinking, emotional blunting
• Acute onset with marked functional decline
• Consistent with age of onset and premorbid personality

**Other Conditions to Consider**

**Acute and transient psychotic disorder (F23)**
o Acute onset with a clear precipitating factor
o To be determined through longitudinal observation

**Substance-induced psychotic disorder (F1x.5)**
o Pending results of drug screening

**Mood disorder with psychotic features**
o Depressive symptoms require further assessment

**Organic mental disorder**
o Ruled out by imaging and laboratory findings

**Treatment Plan**

**Acute Phase Treatment**

**Pharmacotherapy**
o Risperidone 1 mg/day, once daily after dinner
o Monitoring for side effects: Extrapyramidal symptoms, metabolic adverse effects
o Efficacy evaluation: After 2 weeks

**Environmental Adjustment**
o Consideration of hospitalization (to be discussed with family)
o Provision of a low-stimulation environment

**Psychoeducation**
o Explanation of the illness to the patient and family
o Explanation of the importance of medication adherence

**Future Plan**

• Outpatient follow-up in 1 week
• Coordination of a meeting with a psychiatric social worker (PSW)
• Consideration of hospitalization if necessary
• Family meeting to be conducted

**Social Aspects**

• Key persons: Parents
• Level of family understanding: Good; cooperative
• Goal of social reintegration: Return to academic activities
• Financial status: Student (supported by parents)

**Special Notes**

• First episode
• Good cooperation from parents
• Medication adherence requires close attention due to lack of insight
• The graduate school entrance examination is considered a major life stressor and precipitating event

**Next Appointment**

**Date:** In 1 week

**Purpose:**
• Symptom assessment
• Side effect monitoring
• Confirmation of medication adherence
• Consideration of hospitalization if necessary

**Documented by:** Dr. ○○○○
**Date of Entry:** XX, 2024

- **Schizophrenia Case (Generated Note)**Model used: *Claude Sonnet4 (Anthropic)*
  Role: *Internist***Patient ID:** 112D35
  **Date of Visit:** XX, 2024
  **Department:** Psychiatry
  **Physician:** XXX

**Patient Information**

• Name: XXX
• Age/Sex: 21-year-old male
• Occupation: University student
• Family structure: Lives with his parents
• Mode of presentation: Brought in by his parents after being taken into protective custody by the police

**Chief Complaint**

“Brought in by his parents due to bizarre behavior” (as reported by the parents)

**History of Present Illness**

Insomnia developed one week prior to the graduate school entrance examination. After the examination, his symptoms rapidly worsened, and this morning he was taken into protective custody by the police after walking around a bench near a train station for approximately three hours while talking to himself. He has had no prior developmental or adaptive problems and is now presenting with an acute onset of psychiatric symptoms.

**Symptom timeline:**
• One week ago: Took the graduate school entrance examination; onset of insomnia
• After the examination: Difficulty engaging in conversation, avoidance of eye contact, emergence of soliloquy
• Today: Abnormal behavior in a public place, taken into police custody, brought to the hospital by his parents

**Mental Status Examination**

**Consciousness and Orientation**

• Consciousness: Clear (estimated)
• Orientation: Requires further detailed assessment

**Appearance and Attitude**

• Paucity of gestures and facial expressions (emotional blunting)
• Fixed staring; does not attempt to make eye contact
• Sudden shift from inappropriate smiling to fearful facial expression (inappropriate affect)

**Thought and Perception**

• Auditory hallucinations: Appears to listen silently to something and nod (behavior suggestive of responding to hallucinations)
• Soliloquy: Short, dialog-like utterances such as “I see” and “That makes sense”
• Sense of being watched: “Am I being watched? Well, that would make sense” (suggestive)

**Language and Communication**

• No response to questions
• Extremely limited spontaneous speech
• Only abrupt single words or short phrases

**Mood and Affect**

• Emotional blunting
• Inappropriate affect (smiling, fearful facial expressions)

**Motivation and Activity**

• Aimless, stereotyped walking (wandering behavior at the station)
• Marked decline in social functioning

**Physical Symptoms and Findings**

• Insomnia: Persistent for one week
• Loss of appetite: “Barely eating anything”
• Weight loss: Mild (as perceived by family)
• Physical examination and tests: No abnormalities on blood biochemistry, brain MRI, or EEG

**Life History and Developmental History**

• Developmental history: No developmental delays or adaptive problems
• Educational history: Adapted well in kindergarten and school; functioning normally as a university student
• Social adjustment: Previously good

**Past Medical and Medication History**

• Past medical history: No major illnesses or injuries
• Current medications: None
• Allergy history: Unknown (requires confirmation)
• Substance use history: To be confirmed (drugs, alcohol, etc.)

**Family History**

• Family history of psychiatric disorders: Requires further detailed inquiry
• Genetic vulnerability: Unknown

**Premorbid Personality and Adjustment**

• Personality: Quiet, serious, introverted
• Academic functioning: Diligent; aspiring to attend graduate school
• Social functioning: Good premorbid adjustment

**Family Observations and Understanding**

• Parents’ observations:
o “He is usually a quiet child.”
o They are confused by the sudden change.
o They are concerned about his inability to engage in conversation and avoidance of eye contact.
• Understanding of onset: They suspect an association with entrance examination stress

**Diagnosis**

**Primary (Provisional) Diagnosis:** F20.9 Schizophrenia, unspecified (ICD-10)
**DSM-5-TR Diagnosis:** 295.90 Schizophrenia spectrum and other psychotic disorders (provisional)

**Rationale for diagnosis:**
• Auditory hallucinations
• Soliloquy and laughing alone (behavioral responses to abnormal experiences)
• Emotional blunting and inappropriate affect
• Marked decline in social functioning
• Acute onset (approximately one week)

**Differential diagnosis:**

Acute and transient psychotic disorder (F23) – most important to consider due to the short duration since onset

Substance-induced psychotic disorder – urine drug screening required

Mood disorder with psychotic features – detailed evaluation of mood symptoms required

Organic psychotic disorder – organic abnormalities already excluded by physical examination

Delusional disorder – evaluation required for the presence of systematized delusions

Schizophreniform disorder – symptom duration between 1 month and less than 6 months

**Severity and Risk Assessment**

• Psychotic symptoms: Moderate to severe
• Risk of self-harm or harm to others: Requires caution (impaired judgment, abnormal behavior)
• Social functioning: Markedly impaired
• Insight: Absent
• Treatment adherence: Difficult (denial of illness)

**Urgency and Consideration of Hospitalization**

**Suitability for outpatient treatment:**
• Family supervision and cooperation are available
• No clear self-harm or violent behavior
• However, there is a history of abnormal behavior in public places
• Hospitalization should be considered if symptoms progress

**Treatment Plan**

**Pharmacotherapy**

• First-line: Atypical antipsychotic
o Start risperidone at 1 mg/day (after dinner)
o Gradual titration while monitoring efficacy and side effects
o Target dose: 2–6 mg/day

• Side effect monitoring:
o Extrapyramidal symptoms (EPS)
o Metabolic side effects (weight gain, impaired glucose metabolism)
o Hyperprolactinemia
o Sedation

**Adjunctive Treatment**

• Hypnotics: Short-term use if necessary
• Anxiolytics: Consider depending on symptoms

**Psychosocial Interventions**

• Family psychoeducation
• Illness education (promotion of insight)
• Gradual support for social reintegration
• Support to improve medication adherence

**Additional Required Tests and Assessments**

Urine drug screening

Detailed psychiatric symptom assessment (use of rating scales such as PANSS)

Cognitive function assessment (e.g., MoCA, MMSE)

Detailed family history interview

**Follow-up Plan**

• Initial follow-up: 3–5 days later (side effect check after initiation of pharmacotherapy)
• Short-term: Weekly until symptoms stabilize
• Mid-term: Every 2 weeks after improvement
• Long-term: Monthly during the maintenance phase

**Special Notes and Points of Caution**

**Emergency response:** Establish a contact system for symptom worsening; prepare for hospitalization if needed

**Family support:**
o Promote understanding of the illness
o Provide guidance on appropriate responses
o Implement measures to reduce family burden

**Social reintegration:**
o Careful decision-making regarding timing of return to university
o Gradual reintegration program

**Pharmacotherapy:**
o Special attention to side effects in young patients
o Explain the need for long-term treatment

**Prognosis counseling:**
o Distinguish the possibility of an acute psychotic episode from schizophrenia
o Emphasize the importance of early intervention

**Future Treatment Goals**

**Short-term Goals (1–3 months)**

• Reduction of psychotic symptoms
• Improvement in sleep and nutrition
• Recovery of basic communication abilities

**Mid-term Goals (3–12 months)**

• Development of insight
• Establishment of medication adherence
• Gradual social reintegration

**Long-term Goals (After 1 year)**

• Recovery of social functioning
• Return to university and continuation of education
• Prevention of relapse

**Next Appointment**

In 3 days, for side effect assessment and symptom evaluation after initiation of pharmacotherapy

**Attending Physician’s Signature:** XXX
**Date of Entry:** XX, 2024

- **Major Depressive Disorder Case (Generated Note)**Model used: *DeepSeek-R1/Qwen-14B (CyberAgent Inc)*
  Role: *Psychiatrist*

**Basic Information**

Patient: Male, 45 years old, office worker
Employment status: Section manager (6 months since promotion)
Current living situation: Lives with wife and child (household of three)

**Chief Complaints and Past History (Onset ≥ 18 months ago)**

**Primary symptoms:**

Sleep–wake disturbance (insomnia)

Careless mistakes (decline in work efficiency)

Somatic symptoms: decreased appetite and weight loss

Psychological impact: depressed mood, loss of interest in hobbies

**Precipitating factors:**

Expansion of job responsibilities (increased pressure and responsibility)

Tendency to internalize stress and overburden himself

**Mental Status Assessment (Modified CAGE-style screening)**

**Depressed mood:**

Persistent low mood

Loss of interest in hobbies and social activities

Mild passive suicidal ideation (“It might be better if nothing existed”), occurring frequently

**Sleep disturbance:**

Nocturnal awakenings 2–3 times per night

Occurring 4–5 days per week

**Agitation / urgency:**

Tendency to seek small, immediate improvements

Impatient with gradual progress

**Psychosocial and Environmental Analysis (Family and Workplace Factors)**

**Family involvement:**

Wife reports increased domestic burden (patient becoming “apathetic,” leading to breakdown of household role-sharing)

Reduced involvement with childcare

Wife experiencing stress displacement due to increased sense of sole responsibility as the caregiver

**Workplace environment:**

Premorbid perfectionistic tendencies (latent vulnerability prior to promotion)

Insufficient evaluation of current collaborative functioning with two direct subordinates

**Differential Considerations for Physical Symptoms**

**Biological screening considerations:**

Blood tests: TSH, thyroid hormones, vitamin B12, folate (to rule out thyroid dysfunction and nutritional deficiency)

Circadian rhythm dysregulation: possible melatonin secretion disturbance

**Substance-related factors:**

Alcohol use denied; liver function evaluation still under consideration

**Treatment Plan (Utilizing National Health Insurance)**

**Pharmacotherapy:**

SSRI (Sertraline 25 mg/day to start → dose increase after 7 days)

Management of intolerance: hypnotics not to be co-prescribed

**Behavioral interventions:**

Reorganization of household responsibilities (guidance provided to wife)

Small-goal setting support (work efficiency improvement program)

**Follow-up and monitoring:**

One-month follow-up: blood tests (thyroid function, nutritional status) + sleep rhythm assessment

**Family Intervention Plan**

**Guidance points for wife:**

Emphasize reduction of domestic burden for the patient

Prioritize maintaining “non-patient” family relationships through time with the child

**Workplace coordination:**

Collaboration with occupational physician to adjust workload

**Supplementary Clinical Observations (Based on Leadership Experience)**

**Comparison with similar age-group occupational maladaptation cases:**

Early detection of self-critical tendencies at first outpatient visit

Decline in self-evaluation following promotion

**Strategies to prevent stress displacement by spouse:**

Psychoeducation for both partners

**Establishment of a long-term treatment foundation:**

Evaluation of lifestyle improvement rate and medication response at 1-month follow-up

**Next Appointment**

Scheduled for 1 month later (pending blood test results)

**Facility Contact:** Request for preparation of family-oriented advisory materials

- **Major Depressive Disorder Case (Generated Note)**Model used: *DeepSeek-R1/Qwen-14B (CyberAgent Inc)*
  Role: *Internist*

**Patient Information**

Name: (Pseudonym), 45-year-old male, office worker
Date of first visit: XX, 20XX
Referral source: Occupational physician
Family structure: Lives with wife and child (household of three)

**1. Chief Complaint and History of Present Illness**

**Onset of symptoms:** Approximately 6 months after promotion to section manager

**Primary symptoms:**

Difficulty initiating sleep → increase in careless mistakes (impact on work performance)

Dizziness, fatigue, and loss of appetite (subjective awareness of weight loss)

**Course of symptoms:**
Initially presented as simple difficulty falling asleep, gradually progressing to generalized fatigue and apathy.

**2. Mental Status Examination**

Depressed mood: Loss of interest in hobbies; no longer engaging in leisure activities

Manic or delusional state: Denied (no agitation, no extreme elevation of mood or increased activity)

Suicidal ideation: Mild (no specific plan)

Sleep disturbance: Difficulty maintaining sleep (frequent nocturnal awakenings)

**3. Physical Symptoms and Interpretative Model**

**Findings required for differential diagnosis:**

Blood tests are needed to rule out vasculitis and thyroid dysfunction (e.g., TSH, anti-thyroid antibodies)

**Differentiation from insomnia and anxiety disorders:**

Psychogenic insomnia due to excessive sense of responsibility is suspected

**4. Life History and Past History**

Environmental factors: Raised in a strict household (father was strict), presumed to have contributed to the development of a perfectionistic personality

Occupational history: Employed at the same company for over 20 years (risk of cumulative stress)

**5. Diagnosis and Treatment Plan**

**Primary diagnosis:** Major depressive disorder (mild to moderate)

**Differential considerations:**

Thyroid dysfunction

Fatigue due to overwork

**Pharmacotherapy:**

Initiation of an SSRI (sertraline), starting at 25 mg/day with gradual titration

**Side effect precautions:**

Monitor for gastrointestinal symptoms and headaches

**Psychotherapy:**

Supportive therapy to be conducted once weekly

**Family intervention:**

Practical support strategies proposed to the wife (e.g., redistribution of household tasks, securing time for interaction with the child)

**6. Points of Caution**

Neurology referral: Further evaluation of dizziness (brain MRI suggested)

Key guidance for family: Reduce the impact of the patient’s apathy on household burden

Side effect assessment and treatment response evaluation at 1 month

**7. Next Follow-up**

Review of blood test results (by the end of next week)

Adjustment of pharmacotherapy (dose modification depending on symptom improvement or side effects)

**Explanation of Revisions**

**Expanded differential diagnosis:** The original scenario ended with the patient’s subjective belief that stress was the sole cause. Additional testing for thyroid dysfunction and overwork-related fatigue has been explicitly added.

**Clarification of treatment plan:** Gradual titration from 25 mg and specific side effect precautions have been included.

**Strengthened family collaboration:** The original “family guidance” was translated into more concrete, actionable measures (e.g., redistribution of household responsibilities).

*Note: Further details may be added depending on institutional standards, such as prioritization of laboratory tests and inclusion of insurance billing codes (e.g., use of preventive health screening programs).*

- **Schizophrenia Case (Generated Note)**Model used: *DeepSeek-R1/Qwen-14B (CyberAgent Inc)*
  Role: *Psychiatrist*

**Patient:** 21-year-old male (aspiring to enter graduate school)
**Date of Visit:** Reiwa XX, XX, XX

**Chief Complaint and History of Present Illness**

**Chief complaint:** Acutely altered bizarre behavior, insomnia, and flat or expressionless communication.

**Course:** Symptoms began immediately after taking a graduate school entrance examination. The patient was brought to the hospital after being taken into protective custody by the police.

**Family-Reported Symptom Characteristics**

**Over the past week:**

Insomnia (staying up late at night, excessive daytime sleepiness)

Soliloquy and bizarre behaviors (e.g., pacing repeatedly in front of a train station)

Avoidance of eye contact; fixed staring at a single point

**Family observations:**

Abrupt and marked change in behavior (no similar symptoms in the past)

Decreased appetite and weight loss (reported by mother)

**Physical and Behavioral Findings**

Flat affect, avoidance of eye contact, and prolonged silent staring

Sudden emotional shifts (e.g., inappropriate smiling, fearful expressions)

Verbalization of “feeling watched” and brief, fragmented soliloquies

No physical abnormalities detected (normal blood tests, MRI, and EEG)

**Past History**

The patient is a 21-year-old university student with a serious and diligent personality, aspiring to enter graduate school. He has no history of developmental disorders or maladaptive behavior.

No history of substance use.

**Diagnostic Reasoning**

**Suspected schizophrenia:**
The symptom profile is consistent with psychotic features that are typically responsive to atypical antipsychotics (e.g., risperidone), although treatment resistance remains to be evaluated.

**Differential diagnoses considered:**

**Bipolar disorder:** The abrupt mental changes and late-night wakefulness may suggest a manic or hypomanic state.

**Stress response (adjustment disorder):** Environmental stress related to the graduate school entrance examination may have acted as a trigger.

**Treatment Plan**

**Pharmacotherapy**

Initiate risperidone 0.5 mg/day (with careful monitoring for side effects)

Concomitant use of hypnotics is avoided due to potential drug–drug interactions with antipsychotics

**Behavioral and Psychosocial Interventions**

Ensure regular mealtimes with family support to maintain nutritional status

Temporarily suspend plans for graduate school entry and coordinate with the university counseling office

**Follow-Up Monitoring Points**

Blood test results at 1 month (to rule out thyroid dysfunction, malnutrition, and infectious causes)

Progress in re-establishing a regular sleep–wake rhythm

Maintenance of family relationships (psychoeducation for family members prioritized)

**Family Guidance**

Establish a shared family mealtime routine (provide alternative strategies if the patient is unable to initiate this independently)

Develop a family-based suicide risk monitoring system (recommend direct questioning such as, “Have you thought about killing yourself recently?”)

**Next Appointment**

Scheduled for 1 month later, for blood test review and assessment of medication efficacy.

**Important Notes**

This diagnosis is not final and remains provisional pending reevaluation.

It is necessary to distinguish between adolescent-specific self-critical tendencies and true psychotic symptoms.

**Additional revisions:**
Questions regarding family stress burden (e.g., “Have there been recent changes in your relationship with your parents?”) and direct suicide risk assessment with the patient were missing in the original scenario and have now been added.

- **Schizophrenia Case (Generated Note)**Model used: *DeepSeek-R1/Qwen-14B (CyberAgent Inc)*
  Role: *Internist*

**Patient ID:** 112D35
**Sex:** Male
**Age:** 21 years

**Basic Information**

Place of origin: Urban area
Educational background: University student aspiring to enter graduate school
Marital status / children: None

**Reason for Presentation**

One week ago, after taking an entrance examination, the patient developed persistent nighttime insomnia.
This morning, he was found wandering around a bench near a train station for approximately three hours while talking to himself, and was brought to the hospital after being taken into protective custody by the police.

**Chief Complaints Reported by Family**

**Father:**
“Immediately after the exam, he suddenly began exhibiting more bizarre behavior. He is usually quiet, but now he is expressionless and unable to engage in conversation. He also has insomnia and reduced appetite.”

**Mother:**
“He was functioning normally until a week ago, but then suddenly changed. There has been no noticeable change in his relationship with family members.”

**Detailed History of Present Illness**

Nighttime sleep duration: 0 hours per day
Food intake: Approximately 50% reduction (reported by mother)
Weight change: “Slightly decreased,” according to both parents

**Observed Psychiatric Symptoms**

Gestures and facial expressions: Almost no variation; avoids eye contact and appears to stare fixedly at a single point

Conversational response:
Silent in response to questions → suddenly makes brief remarks such as “I see” or “That makes sense”

Non-goal-directed behavior:
Wanders around despite being aware of the presence of security personnel

**Physical Symptoms**

Pain, headache, or other physical discomfort: Denied
Body temperature, blood pressure, etc.: Within normal range (consistent with laboratory results)

**Developmental and Family History**

No history of developmental disorders.
Excellent academic performance and no problems with school progression.

Family environment: Good family relationships; parents are supportive of the patient.
Past major stressors: None

**Diagnostic Considerations**

**Provisional Diagnosis: Schizophrenia-like state (suspected psychotic condition)**

**Rationale:**

Acute hallucination-like experiences (sense of being “monitored” by police officers)

Thought disturbance (soliloquy, asynchronous dialogue)

Impaired volitional control (sudden changes in behavior patterns)

**Differential Diagnosis**

**Anxiety-related syndromes:** Inconsistent due to prolonged course and lack of subjective distress

**Adjustment disorder:** Environmental stress (entrance examination) may have acted as a trigger (family reports that his baseline personality was unchanged)

**Treatment Plan**

**Acute Phase Management**

Risperidone 0.5 mg/day (initiation with careful monitoring for side effects)

Avoid co-administration of hypnotics (considering the possibility of bipolar disorder)

**Behavioral and Psychosocial Interventions**

Family support to ensure regular mealtimes (monitoring nutritional status)

Stress management guidance related to graduate school aspirations

**Follow-up Monitoring Points**

Blood test results at 1 month (thyroid function, nutritional deficiency)

Progress in re-establishing sleep–wake rhythm

Maintenance of family relationships (psychoeducation for spouse and children indicated)

**Advice for the Family**

Create an environment in which the family can share mealtimes together (offer alternatives if the patient is unable to initiate this himself)

Propose collaboration with the university’s student counseling services to temporarily postpone graduate school plans

**Next Appointment**

Scheduled for 1 month later

**Experience-Based Modifications**

Early identification of “self-critical tendencies” in adolescence (to be distinguished from schizophrenic symptoms)

Explicitly listing “sleep medications” in the contraindicated drug list (due to potential interactions with antipsychotics)

**Important Note**

This diagnosis is not yet definitive. Final diagnostic determination will be made at the 1-month re-evaluation.

- **Major Depressive Disorder Case (Generated Note)**Model used: *ELYZA/LLaMA3-8B (ELYZA Inc)*
  Role: *Psychiatrist*

**Patient Information**

Name: (Pseudonym), 45-year-old male
Occupation: Office worker (section manager)
Address: [Unknown]
Contact information: [Unknown]

**History of Present Illness**

Since around mid-2023, the patient has had increasing difficulty initiating sleep and has begun making frequent careless mistakes at work. He has also developed dizziness, general fatigue, and loss of appetite. He presented to the clinic upon the recommendation of the company’s occupational physician.

**Chief Complaint**

“Recently, I have been having trouble falling asleep, and this has continued, leading to an increase in mistakes at work.”

**Past Medical History**

None of note.

**Family History**

His father was strict and very harsh about mistakes.

**Personal and Developmental History**

He was raised in a typical household, and there were no problems with development during childhood. He graduated from university and has been working at his current company ever since.

**Physical Symptoms**

Dizziness, fatigue, and loss of appetite.

**Psychiatric Symptoms**

Depressed mood, mild passive suicidal ideation, feelings of apathy, decreased concentration, nighttime insomnia, and a persistent sense of not feeling rested.

**Treatment Policy**

An antidepressant will be initiated at a low dose and adjusted as needed. Psychotherapy and stress management will be provided concurrently.

**Medication Plan**

Sertraline 50 mg/day (starting at 25 mg/day).

**Next Appointment**

[Unknown]

**Clinical Impression**

A depressive state is the predominant symptom, and stress is considered to be a major contributing factor. Mild passive suicidal ideation is also present, indicating the need for early intervention.

**Physician’s Comment**

Taking into account the patient’s current workplace environment and family circumstances, the physician proposes establishing a structured support system for both the patient and his family.

- **Major Depressive Disorder Case (Generated Note)**Model used: *ELYZA/LLaMA3-8B (ELYZA Inc)*
  Role: *Internist*

**Patient Information**

Name: 45-year-old male
Date of birth: Month ○, 1977
Address: ○○ Ward, ○○ City
Occupation: Office worker (section manager)

**Chief complaint:**
Difficulty falling asleep, increasing careless mistakes at work, accompanied by dizziness, fatigue, and loss of appetite.

**Past Medical History**

None of note.

**Family History**

No family members with similar symptoms.

**Life History and Personality**

He has a strong sense of responsibility and tends to be perfectionistic.
There were no problems with development during childhood.

**Physical Findings and Test Results**

No remarkable physical abnormalities were observed.

**Psychiatric Symptoms and Diagnosis**

A depressive state is the primary clinical feature, and stress is considered to be a major contributing factor.
Mild passive suicidal ideation is also present; therefore, early intervention is required.

**Treatment Plan**

An antidepressant (SSRI) will be initiated at a low dose and adjusted as needed.
Psychotherapy and stress management will be implemented concurrently.

**Instructions and Explanations**

Sertraline 50 mg/day is proposed. After confirming tolerability and the absence of significant side effects, the dose will be adjusted as necessary.
The importance of family support was explained, and understanding was obtained from both the patient and his family.

**Next Appointment**

Follow-up visit scheduled in one week.

**Additional Notes and Precautions**

The patient and his family should be educated about depressive symptoms and suicidal ideation. Suicide risk should be assessed continuously, with regular follow-up visits.
During treatment, attention should be paid to the interactions between the patient and his family. Clinical issues and challenges should be identified, and the treatment plan should be adjusted accordingly.

- **Schizophrenia Case (Generated Note)**Model used: *ELYZA/LLaMA3-8B (ELYZA Inc)*
  Role: *Psychiatrist*

**Patient:** 112D35, 21-year-old male, university student

**Chief Complaint**

Insomnia and bizarre behavior.

**History of Present Illness**

The patient has been experiencing persistent insomnia since taking a graduate school entrance examination one week ago. Recently, meaningful conversation has become difficult, and he avoids making eye contact. He sometimes speaks to himself abruptly and shows sudden changes in affect, such as inappropriate smiling or appearing frightened.

**Past Medical History**

None. His life history, medication history, educational background, and occupational history are all unremarkable.

**Family History**

None.

**Mental Status**

Schizophrenia is considered a possible diagnosis. The patient presents with insomnia, symptoms suggestive of auditory hallucinations, and bizarre behavior. He avoids eye contact and remains mostly silent. At times, he appears to be listening to something and nods, as if responding to internal stimuli.

**Treatment Plan**

Pharmacotherapy will be initiated with risperidone 1 mg/day, with careful monitoring for side effects and dose adjustment as needed. Psychosocial support will be provided concurrently.

**Family’s Requests**

The family wishes for the patient to calm down as soon as possible and asks for guidance on what they should do to help.

- **Schizophrenia Case (Generated Note)**Model used: *ELYZA/LLaMA3-8B (ELYZA Inc)*
  Role: *Internist*

**Patient Information**

Name: 112D35
Age/Sex: 21-year-old male
School/Occupation: University student

**Chief Complaint:**
Inability to sleep at night and bizarre behavior.

**History of Present Illness**

One week ago: Took a graduate school entrance examination.

Since the examination: Persistent insomnia and onset of bizarre behavior.

Recent condition: Talking to himself, avoiding eye contact, and inability to engage in meaningful conversation.

**Physical Findings**

No remarkable physical abnormalities.

**Test Results**

Blood biochemistry: Within normal limits

Brain MRI and EEG: No abnormalities

**Diagnosis and Treatment Plan**

**Diagnosis:**
Possible schizophrenia.

**Treatment plan:**
Pharmacotherapy will be initiated with risperidone 1 mg/day. Psychosocial support will be provided concurrently.

**Family Support**

The importance of family support was explained, and cooperation from the family was obtained.

**Appendix D. Supplementary Figures and Tables**


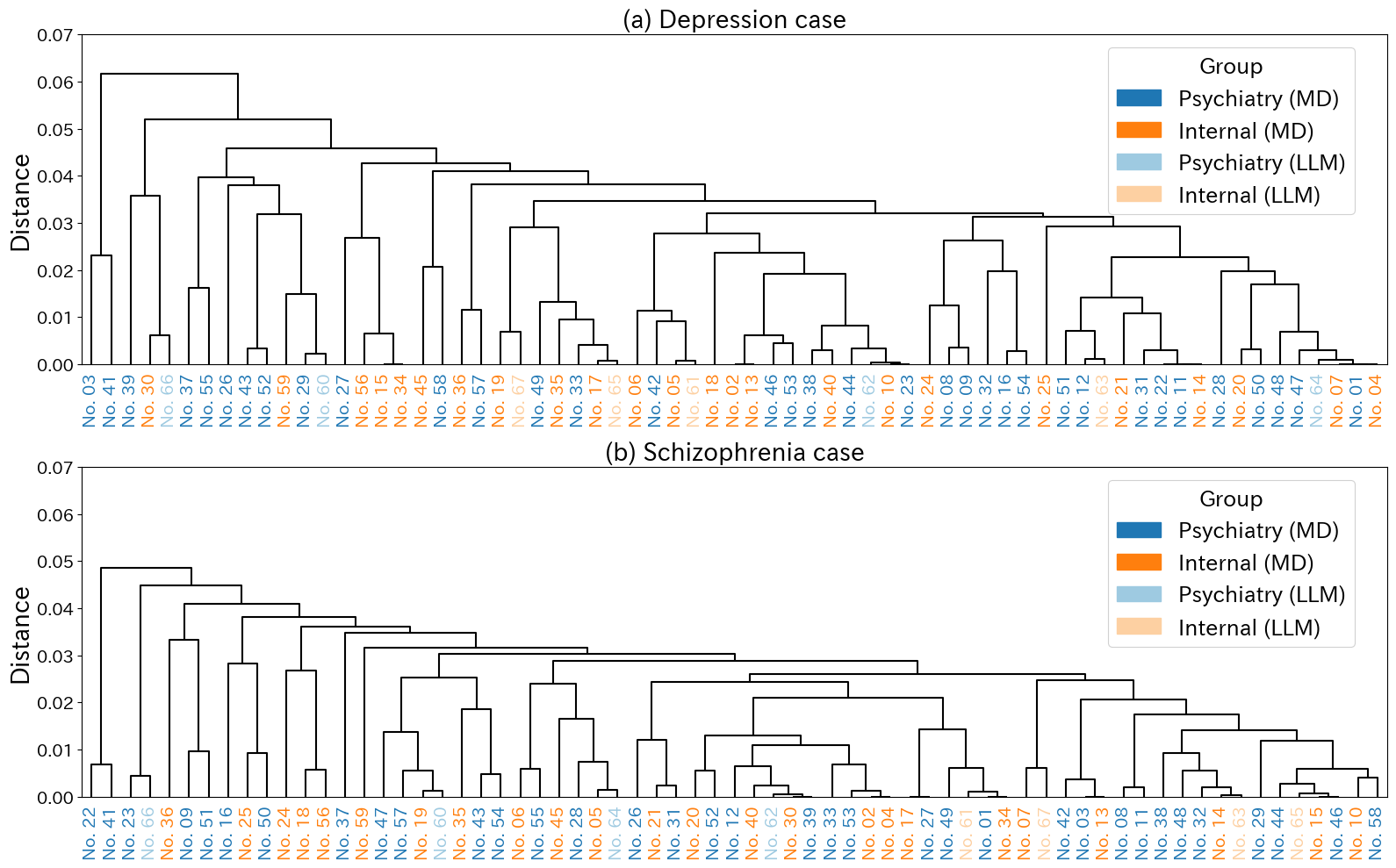


Figure S1. Hierarchical clustering of clinical notes based on syntactic similarity (BLEU) for the depression case (a) and schizophrenia case (b).


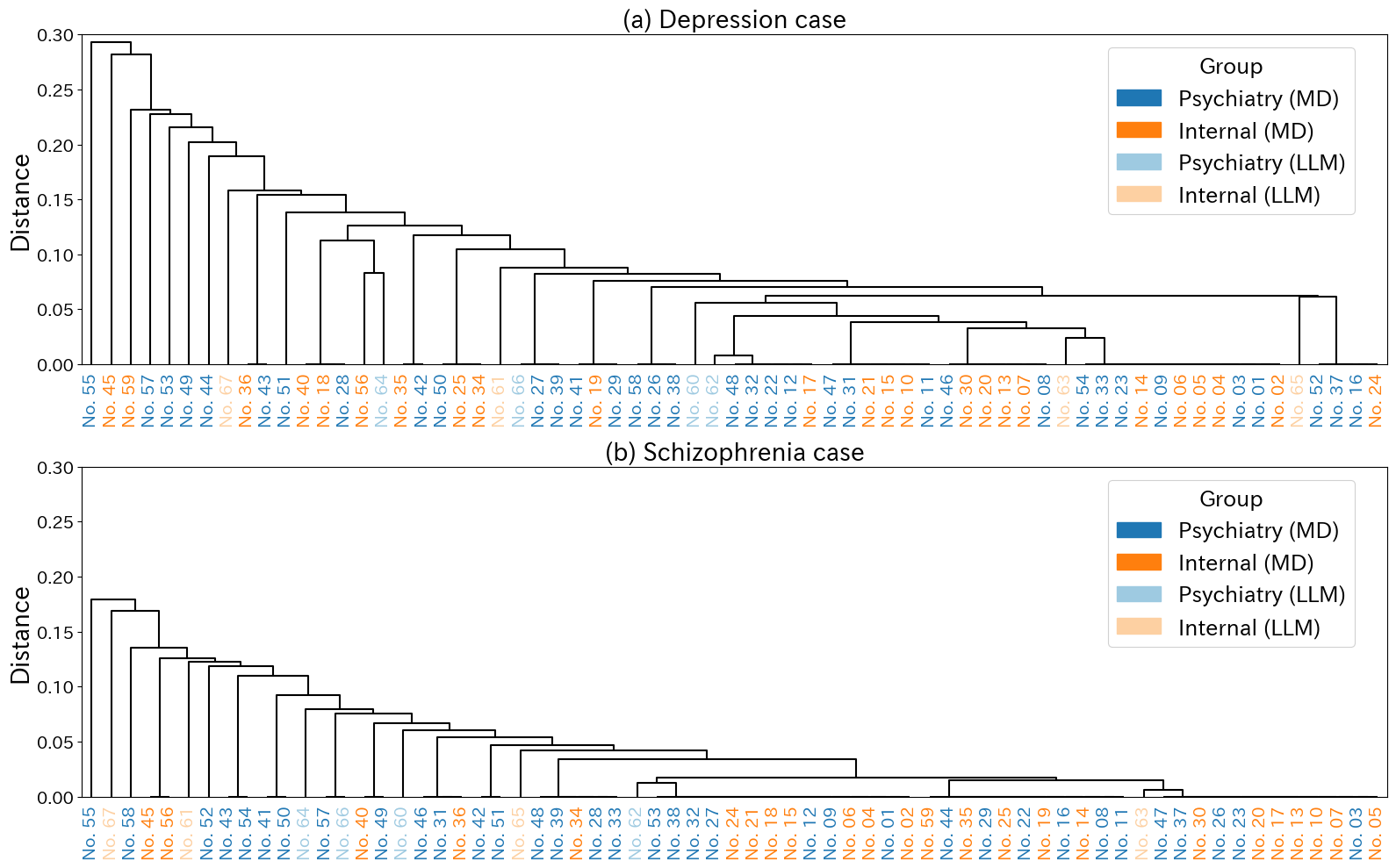


Figure S2. Hierarchical clustering of clinical notes based on syntactic similarity (ROUGE-L) for the depression case (a) and schizophrenia case (b).


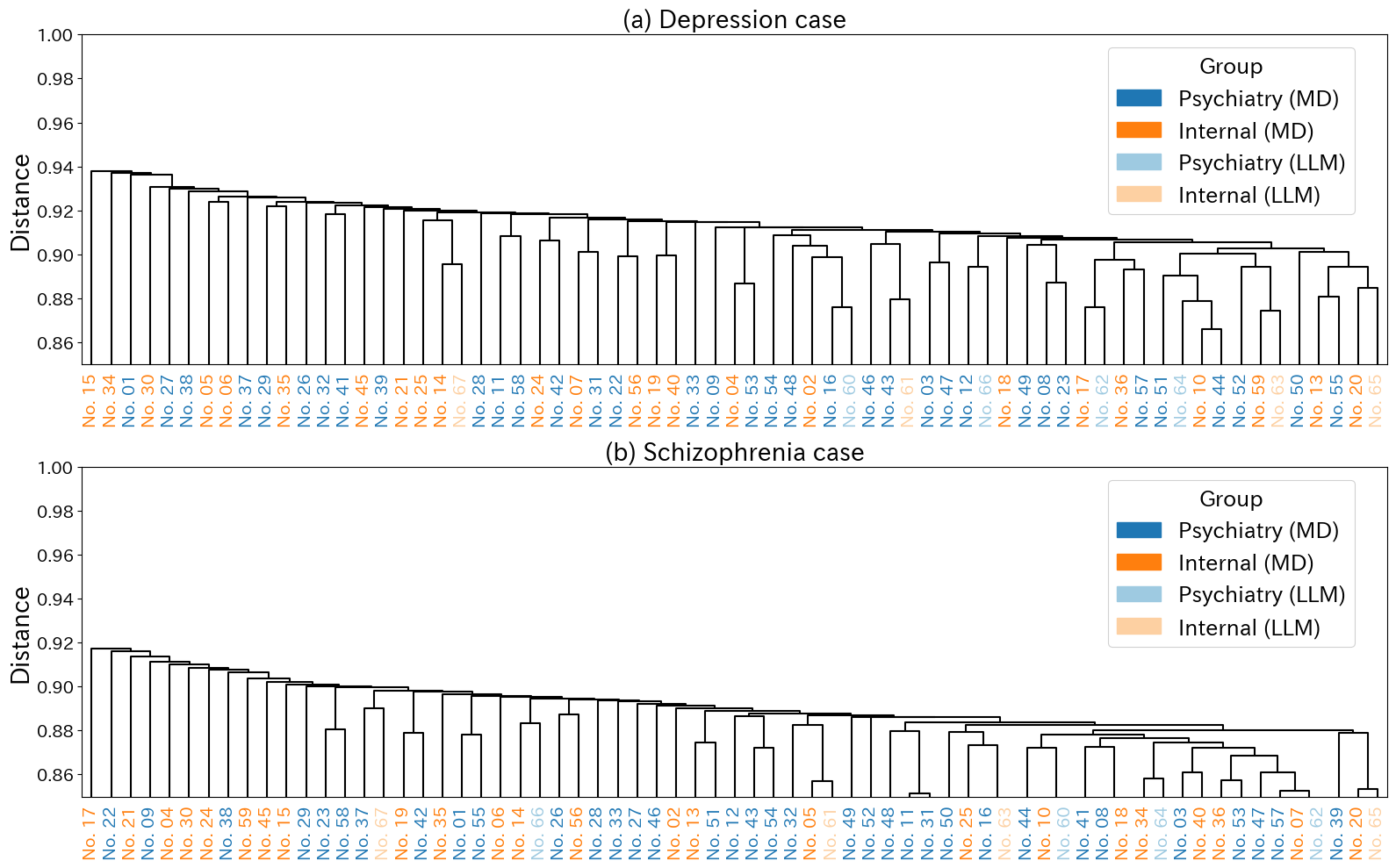


Figure S3. Hierarchical clustering of clinical notes based on semantic similarity (BERTScore) for the depression case (a) and schizophrenia case (b).

Table S1. Top TF-IDF–weighted terms by group for the schizophrenia case.

| **Psychiatrists (Word)** | **TF-IDF** | **Internists (Word)** | **TF-IDF** | **LLM Psychiatry (Word)** | **TF-IDF** | **LLM Internal (Word)** | **TF-IDF** |
| --- | --- | --- | --- | --- | --- | --- | --- |
| Mental | 0.080 | Schizophrenia | 0.141 | None | 0.154 | Family | 0.162 |
| Schizophrenia | 0.070 | Initiation | 0.079 | Family | 0.147 | None | 0.139 |
| Initiation | 0.067 | Male | 0.075 | Mental | 0.141 | Mental | 0.133 |
| Behavior | 0.064 | Suspected | 0.078 | Symptoms | 0.099 | Symptoms | 0.117 |
| Insomnia | 0.060 | Typical | 0.071 | University | 0.113 | Physical | 0.106 |
| Examination | 0.058 | 21 | 0.066 | Etc. | 0.093 | University | 0.100 |
| Risperidone | 0.055 | Risperidone | 0.065 | Mental | 0.085 | Diagnosis | 0.095 |
| Acute | 0.050 | Oral medication | 0.058 | Facial expression | 0.082 | Behavior | 0.088 |
| Auditory hallucination | 0.050 | Abnormal | 0.057 | Strange | 0.082 | Exam | 0.087 |
| Peculiar | 0.048 | Mental | 0.057 | Findings | 0.081 | Treatment | 0.086 |
